# The metallophosphoesterase Rv0805 regulates carbon flux and cell envelope homeostasis during growth of mycobacteria in propionate

**DOI:** 10.1128/jb.00571-25

**Published:** 2026-02-27

**Authors:** Priyanka Biswas, Nishad Matange, Sintu Samanta, Vishwas Mishra, Gerald Larrouy-Maumus, Sandhya S. Visweswariah

**Affiliations:** 1 Department of Developmental Biology and Genetics, Indian Institute of Science 29120, Bengaluru, India; 2 Department of Life Sciences, Imperial College London 4615, London, United Kingdom; 3 Department of Infectious Disease, Imperial College London 4615, London, United Kingdom; University of Notre Dame, Notre Dame, Indiana, USA

**Keywords:** propionate metabolism, cell wall integrity, *Mycobacterium tuberculosis*, methylcitrate cycle, Rv0805 metallophosphoesterase

## Abstract

*Mycobacterium tuberculosis* relies on host-derived lipids, including cholesterol, for intracellular survival, generating propionyl-CoA—a metabolite that must be efficiently assimilated to prevent toxicity. The metallophosphoesterase Rv0805 is required for optimal growth on cholesterol, and an *Rv0805* knockout strain exhibits impaired ability to colonize the murine lung. However, the mechanisms underlying the essential role of Rv0805 under host-relevant conditions remain unclear. The deletion of the *rv0805* ortholog (*bcg_0857*) in *Mycobacterium bovis* BCG reveals that both its catalytic activity and membrane localization are essential for growth on propionate, a by-product of cholesterol metabolism. Loss of Rv0805 impaired propionate uptake, altered cell envelope lipid composition with an accumulation of methyl-branched lipids, and reduced carbon flux through the methylcitrate cycle, ultimately depleting key central carbon metabolites required for growth. Vitamin B_12_ supplementation activated the methylmalonyl pathway, restoring metabolic balance and rescuing growth. These findings demonstrate that Rv0805 links propionate metabolism with cell envelope integrity, identifying its activity and localization as metabolic vulnerabilities that could be exploited for tuberculosis therapy.

**IMPORTANCE:**

Rv0805 links propionate metabolism with cell envelope homeostasis in mycobacteria, and its loss uncovers a metabolic vulnerability that could be exploited to restrict mycobacterial survival in lipid-rich host microenvironments.

## INTRODUCTION

*Mycobacterium tuberculosis* (*Mtb*) infection causes human tuberculosis which remains the leading cause of death with an estimated 1.25 million deaths and 10.8 million new infections in 2023 (1). *Mtb* inhabits the hostile environment of macrophage phagosomes, exposing them to low pH, hypoxia, reactive oxygen and nitrogen species, and nutrient limitation (2, 3). However, *Mtb* undergoes metabolic adaptations crucial for its survival and pathogenesis (4–6). Deciphering the metabolic strategies employed by *Mtb* is, therefore, imperative for identifying new antimycobacterial strategies.

Mycobacteria possess remarkable metabolic flexibility, which facilitates survival in intracellular environments by co-utilizing multiple carbon sources in a compartmentalized manner to support efficient growth (5, 7, 8). Importantly, *Mtb* shifts toward host-derived lipids, including fatty acids and cholesterol as major carbon substrates during infection (9–11). These substrates not only fuel central carbon metabolism (CCM) but also provide building blocks for virulence-associated cell envelope lipids, which modulate host immunity and support pathogenic persistence (9, 12).

*Mtb* utilizes cholesterol as a carbon source, which confers an evolutionary advantage in mammalian hosts, where an abundant supply of cholesterol is available (13). Cholesterol is broken down to yield four propionyl-CoA, four acetyl-CoA, one pyruvate, and one succinyl-CoA (14), which feed into CCM, fueling energy production, biosynthesis, and anaplerosis (10, 15–17). *Mtb* prominently produces propionyl-CoA from odd-chain fatty acids and branched-chain amino acids during infection. Propionyl-CoA is converted to succinate either via the vitamin B_12_-independent methylcitrate cycle (MCC) (13, 18, 19) or the vitamin B_12_-dependent methylmalonyl (MM) pathways (17, 20). In the absence of vitamin B_12_, MCC becomes the predominant route for anaplerosis from propionate as mycobacteria are unable to synthesize this cofactor (18, 21). However, recent evidence from murine and human infection models indicates that sufficient host-derived vitamin B_12_ is available *in vivo* to activate the MM pathway, rendering MCC largely dispensable during infection (22). Propionyl-CoA detoxification is also possible via conversion to methylmalonyl-CoA (MM-CoA), followed by incorporation of MM-CoA precursor molecules into the methyl-branched lipids. Thus, during infection, mycobacteria utilizing cholesterol harbor elevated levels of phthiocerol dimycocerosates (PDIMs), sulfolipid (SL), and phenolic glycolipids (PGLs) as excess propionyl-CoA derived from cholesterol is shunted toward the biogenesis of these methyl-branched lipids (20). Similarly, the methyl-branched acyl chains of PDIMs and SL become longer as they assimilate cholesterol or propionate. This metabolic flexibility around propionyl-CoA is central to *Mtb*’s ability to maintain efficient CCM and mitigate cholesterol-derived metabolic stress (9, 19).

In our earlier studies on the role of cAMP in mycobacteria, we identified Rv0805 as a cyclic nucleotide phosphodiesterase (23). Rv0805 is a member of the metallophosphoesterase family (24), and structural studies elucidated the conservation of the metallophosphoesterase fold and identified critical residues required for catalytic activity (25). Rv0805 orthologs are only found in the genomes of slow-growing and pathogenic mycobacteria (23). While Rv0805 does hydrolyze cAMP, it does so with low affinity and can exhibit phosphodiesterase (PDE) activity against other cyclic and linear substrates *in vitro* (23, 25, 26). The overexpression in *Mtb* leads to only modest (~30%) reduction in intracellular cAMP levels and triggers phenotypes that appear independent of its cAMP hydrolase activity (27). This functional promiscuity suggests broader physiological roles beyond the canonical cAMP signaling pathway.

Rv0805 adopts the conserved αββα-MPE fold with a binuclear Mn^2+^/Mg^2+^ active site coordinated by residues including Asp97 (N97), which is essential for PDE activity (23, 25, 26). Substitution of Asp97 with alanine (N97A) abolishes catalytic activity without affecting folding or localization. A unique C-terminal cap domain regulates access to the active site, affecting enzymatic stability, oligomerization, and membrane localization (23, 25, 26). The deletion of the final 40 residues (Rv0805Δ40) disrupts envelope localization and attenuates the enzyme’s physiological functions without affecting catalytic activity (26, 28). When expressed in *Mycobacterium smegmatis* (*M. smegmatis*), the cap domain modulates cell envelope permeability, altering susceptibility to hydrophobic cytotoxic compounds (26, 28).

In a study by Griffin et al., *rv0805* was identified as one of the genes essential for optimum growth of *Mtb* on cholesterol (16, 29). An independent study showed that the deletion of *rv0805* from *Mtb* reduced growth in cholesterol and colonization of the lungs in mice (30). Supplementation with vitamin B_12_ could rescue the growth defect in BCGΔ*rv0805* observed in propionate-containing media*,* and the report suggested that these changes were due to alterations in intracellular cAMP levels (30). However, the mechanistic link between Rv0805 and propionate metabolism, coupled with cell wall localization, remains unanswered.

*Mycobacterium bovis* bacille Calmette-Guérin (BCG), the only licensed vaccine against tuberculosis, is a live attenuated member of the *M. tuberculosis* complex (MTBC) and was derived from *M. bovis* through prolonged *in vitro* passage (31). BCG lacks several virulence-associated regions, including RD1 encoding the ESX-1 secretion system (32, 33). Despite these differences, BCG shares >99% genome sequence identity with *Mtb* and conserves core metabolic pathways essential for intracellular survival, making it a widely used and genetically tractable model for investigating mycobacterial physiology under non-Biosafety Level 3 (BSL-3) conditions (33, 34).

Here, we elucidate the mechanism underlying the role of Rv0805 in propionate metabolism. We show that Rv0805 promotes efficient propionate utilization by maintaining cell envelope lipid homeostasis, which is associated with altered carbon allocation at the propionyl-CoA “node” that intersects the MCC and MM pathways. These findings demonstrate that the loss of function of Rv0805 creates a metabolic vulnerability that could be exploited for the treatment of tuberculosis.

## RESULTS

### Rv0805 is critical for growth in propionate

**Fig 1:**
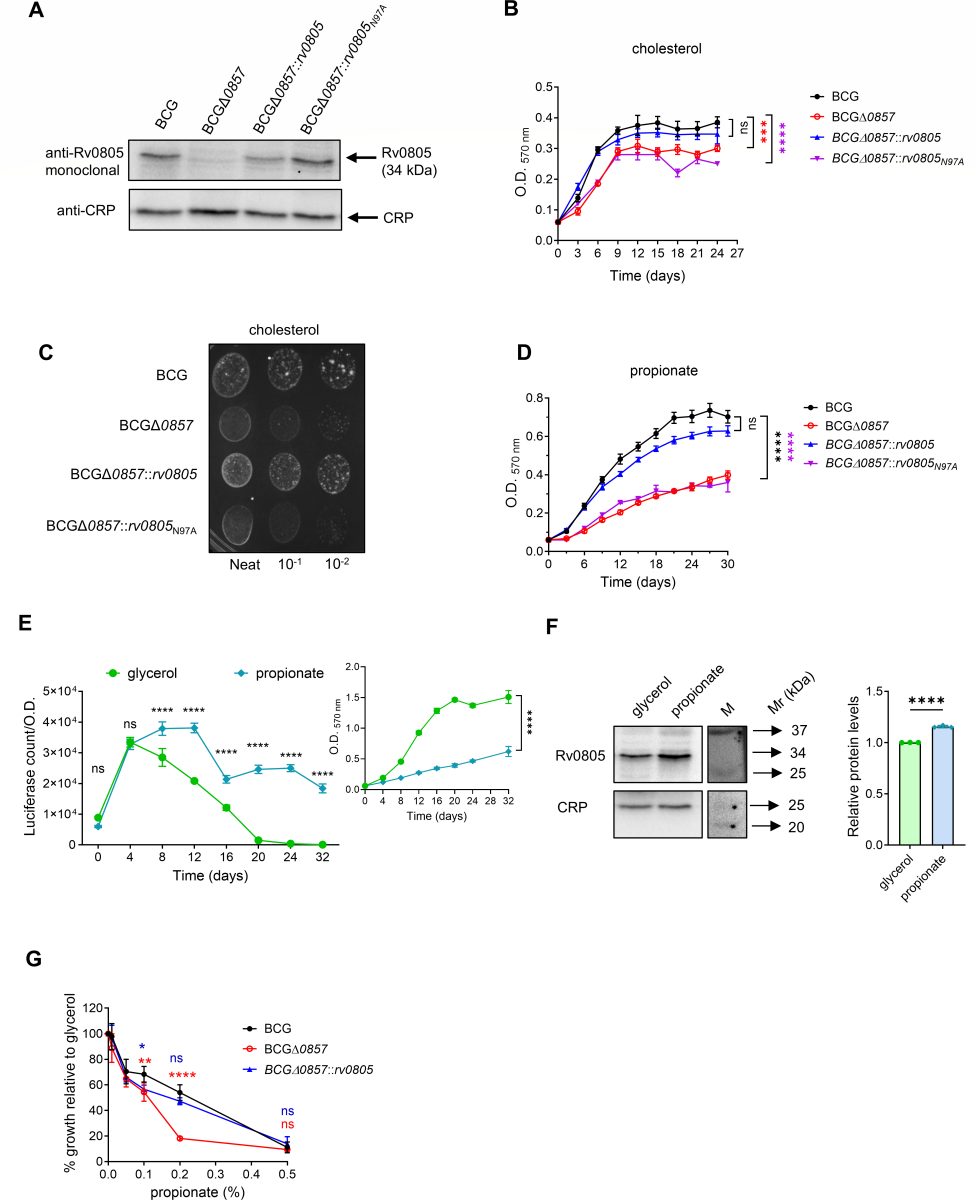
Rv0805 is crucial for growth in propionate. (**A**) Representative Western blot showing the 34 kDa band for Rv0805/BCG_0857 present in BCG, BCGΔ*0857::rv0805*, BCGΔ*0857::rv0805_N97A_,* but not BCGΔ*0857,* grown in propionate (*n* = 2). C-reactive protein (CRP) was used as a normalization control. (**B**) Growth curves in cholesterol minimal media (*n* ≥ 4). (**C**) Growth analysis on cholesterol agar (*n* ≥ 4). (**D**) Growth curves in propionate minimal medium (*n* ≥ 4). (**E**) *bcg_0857* promoter activity in modified wild-type BCG (BCG_WT_*rv0805prom_luxA*) grown in glycerol or propionate minimal media (*n* = 4). The inset shows the growth of the same strain in glycerol or propionate. (**F**) Representative Western blot showing the 34 kDa BCG_0857 protein in exponential phase BCG cultures grown in glycerol or propionate minimal media (*n* = 3) and its quantification. (**G**) Determination of sensitivity to propionate-induced toxicity: growth analysis in glycerol minimal media supplemented with increasing concentrations of propionate (*n* = 4). For **B, D–G**, results show mean ± SEM from biological replicates. Two-way ANOVA determined statistical significance with Dunnett’s multiple comparison test for **B and D**; by two-way ANOVA with Sidak’s multiple comparison test for **E and G**; and by two-tailed unpaired *t*-test for **F**. *P < 0.05; **P < 0.01; ***P < 0.001; ****P < 0.0001; ns, not significant.

To validate earlier reports demonstrating a requirement of Rv0805 during mycobacterial growth on propionate (30), we deleted *bcg-0857* from wild-type *Mycobacterium bovis* BCG. Rv0805 from *Mtb* and its BCG ortholog BCG_0857 are 100% identical at both the nucleotide and amino acid levels, supporting functional conservation between two members of the *M. tuberculosis* complex. We generated complementation strains with wild-type *rv0805*, the catalytically inactive N97A, or the membrane localization-defective Rv0805Δ40 mutant expressed from the *rv0805* native promoter integrated at the *att* site (Fig. S1A). Western blot analysis confirmed the deletion and the presence of the expected 34 kDa Rv0805/BCG_0857 protein in the complemented strains BCGΔ*0857::rv0805* and BCGΔ*0857::rv0805_N97A_* (Fig. 1A).

We assessed the growth of BCG strains on cholesterol and propionate as the sole carbon source, and as reported earlier (30), BCGΔ*0857* displayed slower growth when either cholesterol or propionate was used as a carbon source (Fig. 1B through D). In contrast to the earlier published report (30), however, a significant growth defect was also observed when glycerol was used as a carbon source (Fig. S2A). *Bcg_0857* promoter activity and protein expression were significantly higher in propionate-grown cells compared to glycerol-grown cells (Fig. 1E and F), indicating a requirement for Rv0805 protein in propionate-containing media. Propionate supplementation to glycerol-containing medium significantly impaired the growth of BCGΔ*0857* (Fig. 1G), suggesting that toxicity is a result of the presence of propionate in the medium.

The BCGΔ*0857::rv0805_N97A_* showed phenotypes similar to those of BCGΔ*0857* (Fig. 1B through D; Fig. S2A) in cholesterol, glycerol, or propionate-containing media, confirming that catalytic activity is essential for Rv0805 function during growth in propionate. Complementation of *BCGΔ0857* with a C-terminal truncation mutant lacking the final 40 residues (Rv0805Δ40) that are required for envelope association (28) did not restore growth (Fig. S2B), confirming earlier observations (30). In contrast, however, no significant differences in intracellular cAMP levels were detected, indicating that the growth defect was not dependent on cAMP signaling (Fig. S2C and D).

Together, these data suggest that Rv0805 plays a crucial role during the growth of *Mycobacterium* during cholesterol metabolism and propionate detoxification.

### Deletion of *bcg_0857* affects propionate uptake via altering cell wall lipid levels

**Fig 2:**
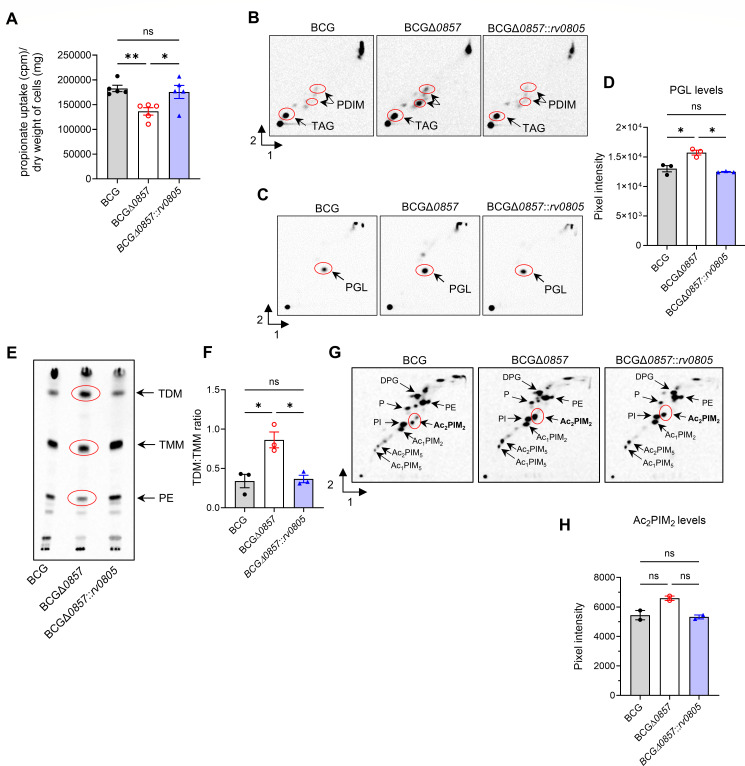
Loss of Rv085 impairs propionate uptake and is associated with altered cell envelope composition. (**A**) ^14^C-propionate uptake in exponential phase propionate-grown cultures (*n* ≥ 4). Representative TLC shows polar/apolar lipid analysis in propionate-grown and 1,2-^14^C sodium acetate radiolabeled exponential-phase cultures (*n* = 3). Accumulation of (**B**) PDIM and (**C**) PGL levels, both derived from propionyl-/methylmalonyl-CoA (*n* = 3). (**D**) Quantification of PGL levels. Levels of (**E**) apolar TDM, TMM, and PE, and (**G**) polar lipids (*n* = 3). Quantification of (**F**) TDM: TMM ratio, and (**H**) Ac_2_PIM_2_ levels. Marked in red are lipids of different intensity among strains. For A, D, F, and H, results show mean ± SEM from biological replicates, and statistical significance was determined by one-way ANOVA with Tukey’s multiple comparison test. *P < 0.05; **P < 0.01; ns, not significant. TLC, thin-layer chromatography; PDIM, phthiocerol dimycocerosate; PGL, phenolic glycolipid; TDM, trehalose dimycolate; TMM, trehalose monomycolate; PE, phosphatidylethanolamine; DPG, diphosphatidyl glycerol; PIM, phosphatidylinositol mannosides (integers denote number of mannoside or acyl [Ac] groups); PI, phosphatidylinositol; P, phospholipid.

Rv0805 localizes and interacts with the cell membrane and cell wall of mycobacteria (26, 28). We, therefore, asked if the growth defect observed was a direct consequence of altered cell membrane properties and nutrient uptake. Indeed, ^14^C-propionate uptake was significantly lower in BCGΔ*0857* than in BCG or BCGΔ*0857::rv0805* (Fig. 2A). We next analyzed polar and apolar lipids in propionate-grown cells. Despite reduced propionate uptake, BCGΔ*0857* accumulated higher levels of PDIM and PGL that are derivatives of propionyl-CoA and methylmalonyl-CoA than the wild type or complemented strain (Fig. 2B through D). BCGΔ*0857* also displayed an altered TDM/TMM ratio and elevated Ac_2_PIM_2_ levels (Fig. 2E through H), indicating envelope lipid remodeling.

**Fig 3:**
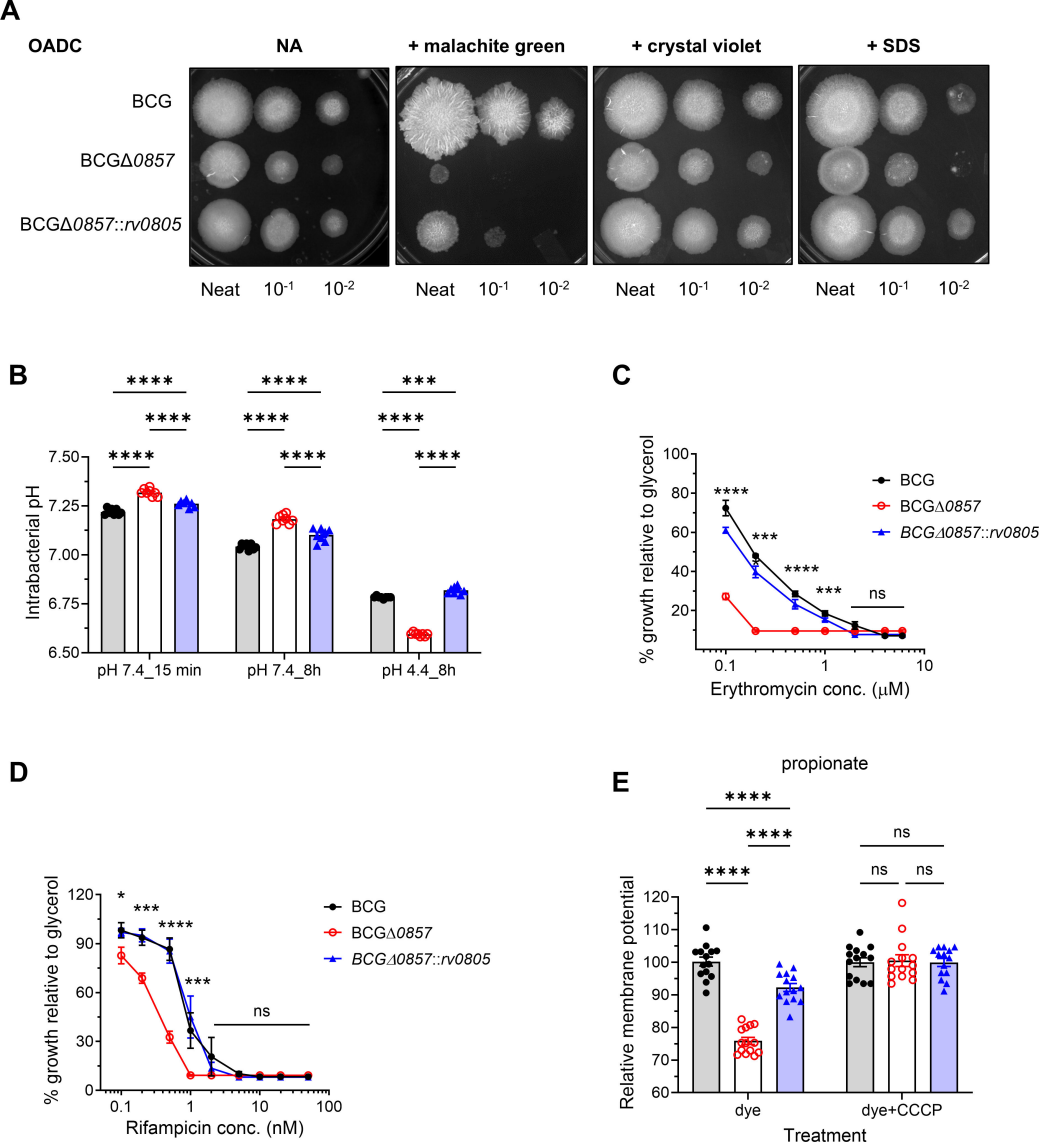
Loss of Rv0805 induces cell envelope defects. (**A**) Representative images of spotting assays on OADC agar ± cell wall-perturbing agents (malachite green, crystal violet, SDS) (*n* ≥ 4). (**B**) Intrabacterial pH of glycerol-grown exponential phase cultures exposed to neutral (pH 7.4) or acidic (pH 4.4) conditions for indicated timepoints (*n* = 8). (**C and D**) Strains were grown in glycerol ± lipophilic antibiotics (erythromycin, rifampicin) (*n* ≥ 2). (**E**) Membrane potential of propionate-grown exponential phase cultures (*n* ≥ 7). CCCP was used as a control. Statistical significance was determined by two-way ANOVA with Tukey’s multiple comparison test for B–D, and by one-way ANOVA with Tukey’s multiple comparison test for F. *P < 0.05; ***P < 0.001; ****P < 0.0001; ns, not significant. CCCP, carbonyl cyanide *m*-chlorophenyl hydrazone.

To assess the functional consequences of altered lipid composition, we tested the susceptibility of these strains to cell wall-perturbing agents, investigated intracellular pH as an indicator of membrane homeostasis, analyzed their sensitivity to lipophilic antibiotics, and estimated the membrane potential in these strains. BCGΔ*0857* showed a higher sensitivity to cell wall-perturbing agents than BCG and BCGΔ*0857::rv0805* (Fig. 3A). Furthermore, BCGΔ*0857* was poorer in maintaining intracellular pH (Fig. 3B). Interestingly, BCGΔ*0857* was more sensitive to the lipophilic antibiotics, erythromycin and rifampicin (Fig. 3C and D). Finally, BCGΔ*0857* exhibited a reduced membrane potential when grown in propionate medium (Fig. 3E). Together, these results suggest a role for Rv0805 in maintaining an optimal lipid profile that preserves membrane integrity, a role that has been previously underappreciated.

### Deletion of *bcg_0857* affects CCM via disrupting carbon flux from propionate

**Fig 4:**
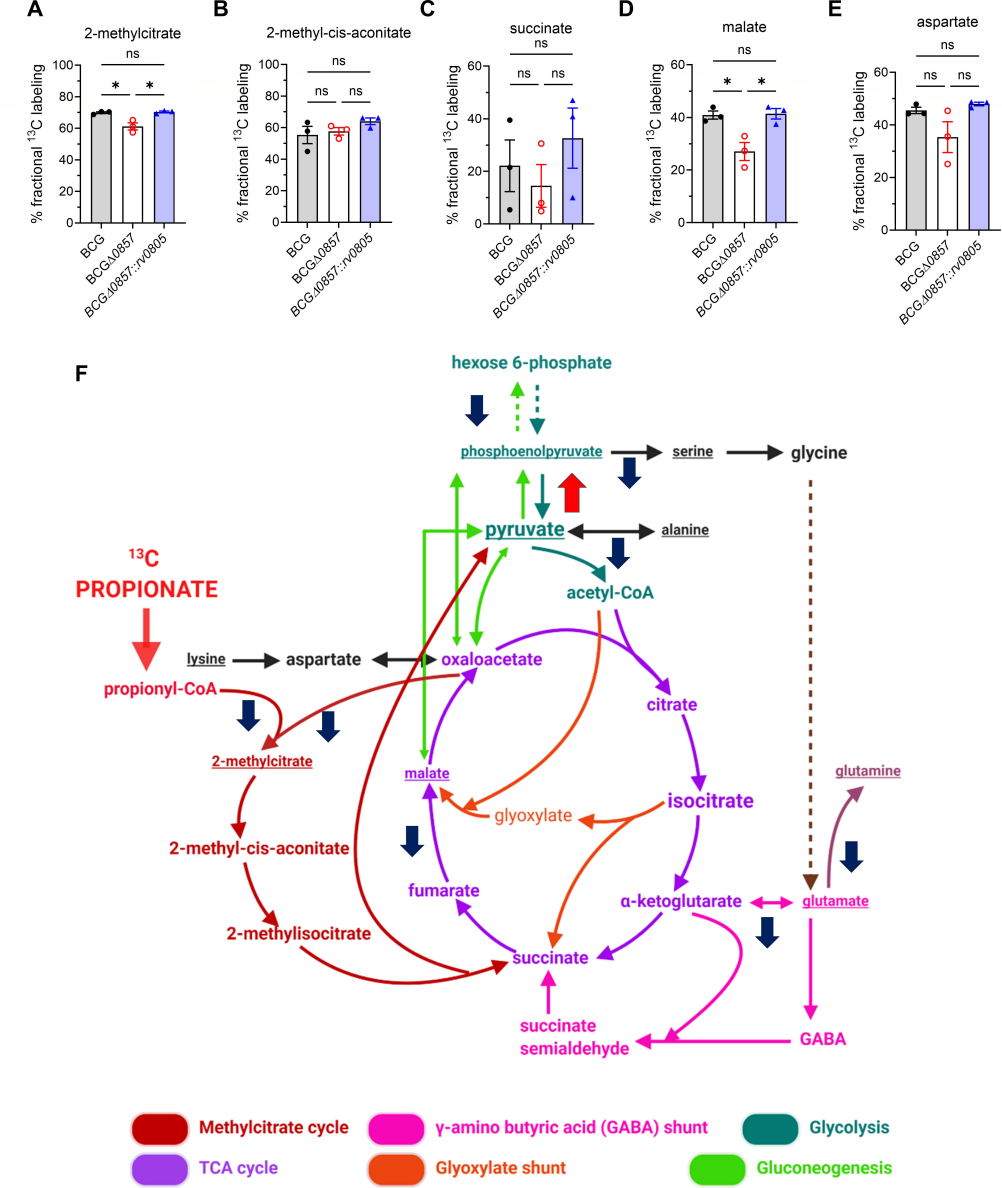
Absence of Rv0805 alters carbon flux into CCM. Fractional ^13^C-labeling in (**A**) 2-methylcitrate, (**B**) 2-methyl-cis-aconitate, (**C**) succinate, (**D**) malate, and (**E**) aspartate from [U-^13^C] propionate (*n* = 3). Results show mean ± SEM from biological replicates. Statistical significance was determined by one-way ANOVA with Tukey’s multiple comparison test, *P < 0.05; ns, not significant. (**F**) Visual representation of impaired ^13^C incorporation into key CCM intermediates. Blue-downward and red-upward arrows indicate lower and higher ^13^C fractional labeling, respectively.

We hypothesized that the reduced propionate uptake and higher PDIM and PGL of the BCGΔ*0857* strain could be due to an altered metabolic flux of propionate. Propionate catabolism converges on CCM via the MCC and MM pathway. ^13^C tracing with [U-^13^C] propionate revealed lower incorporation of ^13^C into 2-methylcitrate and malate in BCGΔ*0857*, while the labeling of 2-methyl-cis-aconitate, succinate, and aspartate remained unchanged (Fig. 4A through F). Similarly, lower ^13^C enrichment was also observed in phosphoenolpyruvate, serine, alanine, glutamate, and glutamine with no differences in glycine, trans-aconitate, isocitrate, and α-ketoglutarate (Fig. S3A through D, F through J). Interestingly, a modest increase in pyruvate labeling, which remained at very low fractional abundance (~1%–3%) (Fig. S3E; Fig. 4F) was seen. Consistent with the increased ^13^C labeling of pyruvate, the anaplerotic node genes (35) *pca*, *ppdk*, and *pckA* were upregulated in BCGΔ*0857* compared to BCG, while *mez* expression remained unchanged (Fig. S3K). These data indicate that the deletion of *bcg_0857* reduces the flux of carbon from propionate into the MCC, impairing CCM.

To determine whether the reduced flux to MCC was a consequence of dysregulated expression of enzymes involved in the pathway, we performed quantitative real-time PCR (qRT-PCR) analysis of the MCC pathway genes. As expected, MCC pathway genes (*prpC*, *prpD*, *icl1*, *icl2*) and their regulator *prpR* (36) were upregulated in both BCG and BCGΔ*0857* in propionate compared to glycerol (Fig. S4A through E). No significant differences in the expression levels of *prpC*, *icl1,* and *prpR* were observed across the strains (Fig. S4A, C and E). However, *prpD* was significantly downregulated, and *icl2* was upregulated in BCGΔ*0857* (Fig. S4B and D). Similarly, other MCC-associated genes encoding SDH1 subunits (*rv0247c*, *rv0248c*, and *rv0249c*), fumarate reductase subunits (*frdABCD*), SDH subunits (*sdhABCD*), fumarase (*fum*), malate: quinone oxidoreductase (*mqo*), and malate dehydrogenase (*mdh*) were expressed at similar or higher levels in BCGΔ*0857* than in BCG (Fig. S4F). Although *prpD* expression was reduced and *icl2* expression increased in BCGΔ*0857*, these opposing transcriptional changes are unlikely to explain the reduced carbon flux through the MCC. Reduced *prpD* expression would be expected to constrain early steps of the pathway, while increased *icl2* expression may reflect a compensatory response. Consistent with reduced ^13^C incorporation into MCC intermediates, the data indicate that impaired propionate assimilation is not primarily driven by altered MCC gene expression.

**Fig 5:**
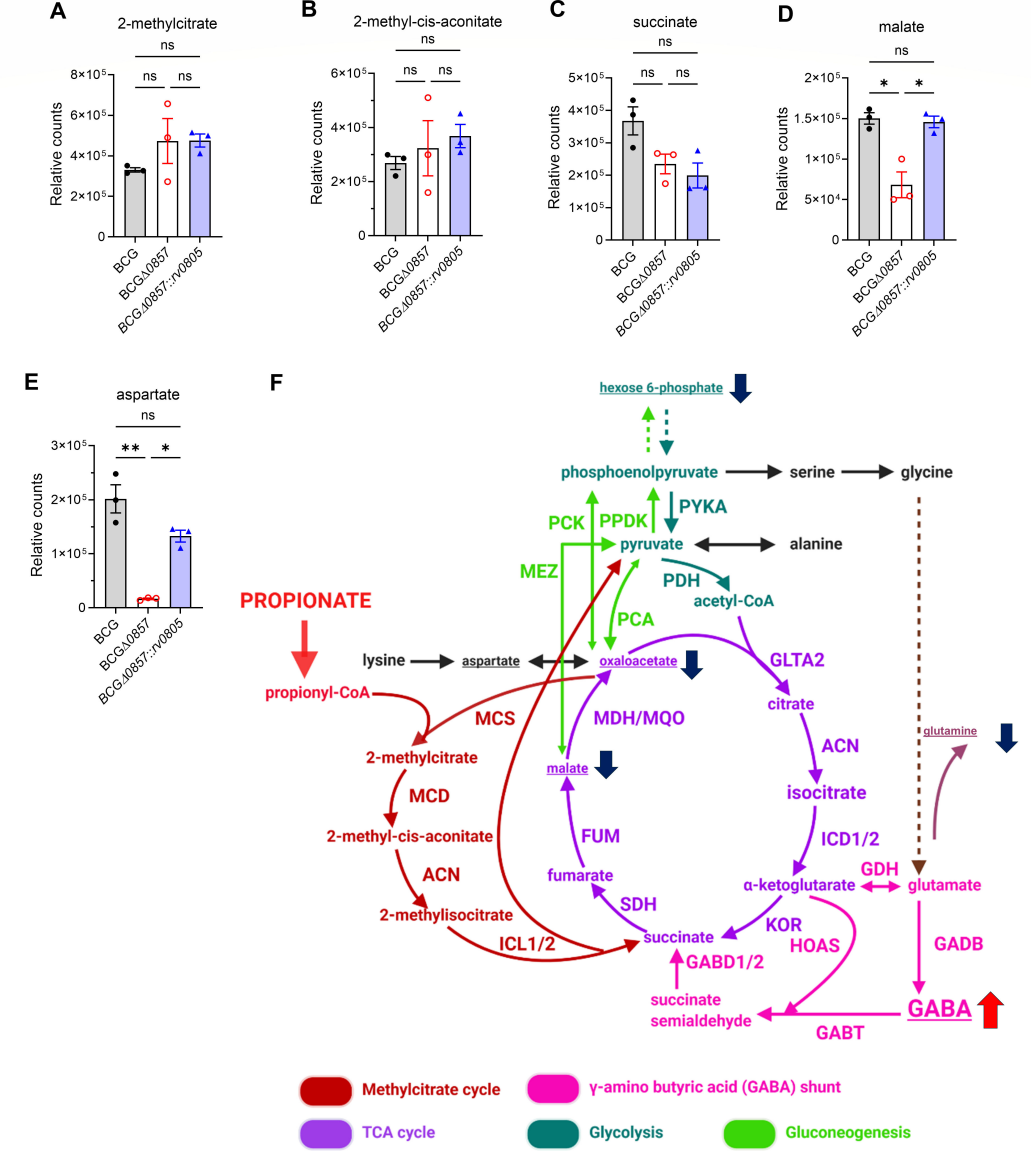
Loss of Rv0805 alters CCM metabolite levels. Steady-state metabolite levels of (**A**) 2-methylcitrate, (**B**) 2-methyl-cis-aconitate, (**C**) succinate, (**D**) malate, and (**E**) aspartate in propionate-grown cultures (*n* = 3). (**F**) Visual representation of impaired CCM in propionate-grown BCGΔ*0857*. Blue down arrows indicate metabolites (underlined) with lower levels, and red up arrows indicate metabolites (underlined) with increased levels in BCGΔ*0857*. For A–E, results show mean ± SEM from biological replicates, and statistical significance was determined by one-way ANOVA with Tukey’s multiple comparison test, *P < 0.05; **P < 0.01; ns, not significant.

To further understand the consequences of the reduced flux into CCM, we profiled metabolites of the MCC. Levels of 2-methylcitrate, 2-methyl-cis-aconitate, and succinate were comparable across strains (Fig. 5A through C); however, BCGΔ*0857* exhibited ~7-fold lower malate and ~36-fold lower aspartate (a proxy for oxaloacetate) (Fig. 5D through F) relative to BCG and BCGΔ*0857::rv0805*. Moreover, while no differences were observed in the levels of other central carbon metabolites—glycine, pyruvate, alanine, aconitate, α-ketoglutarate, and glutamate—BCGΔ*0857* exhibited significantly lower hexose 6-phosphate and glutamine levels, and higher GABA levels than BCG (Fig. S5A through I; Fig. 5F), indicating an altered and impaired CCM.

### Vitamin B_12_ rescues the growth defect by activating the MM pathway

**Fig 6:**
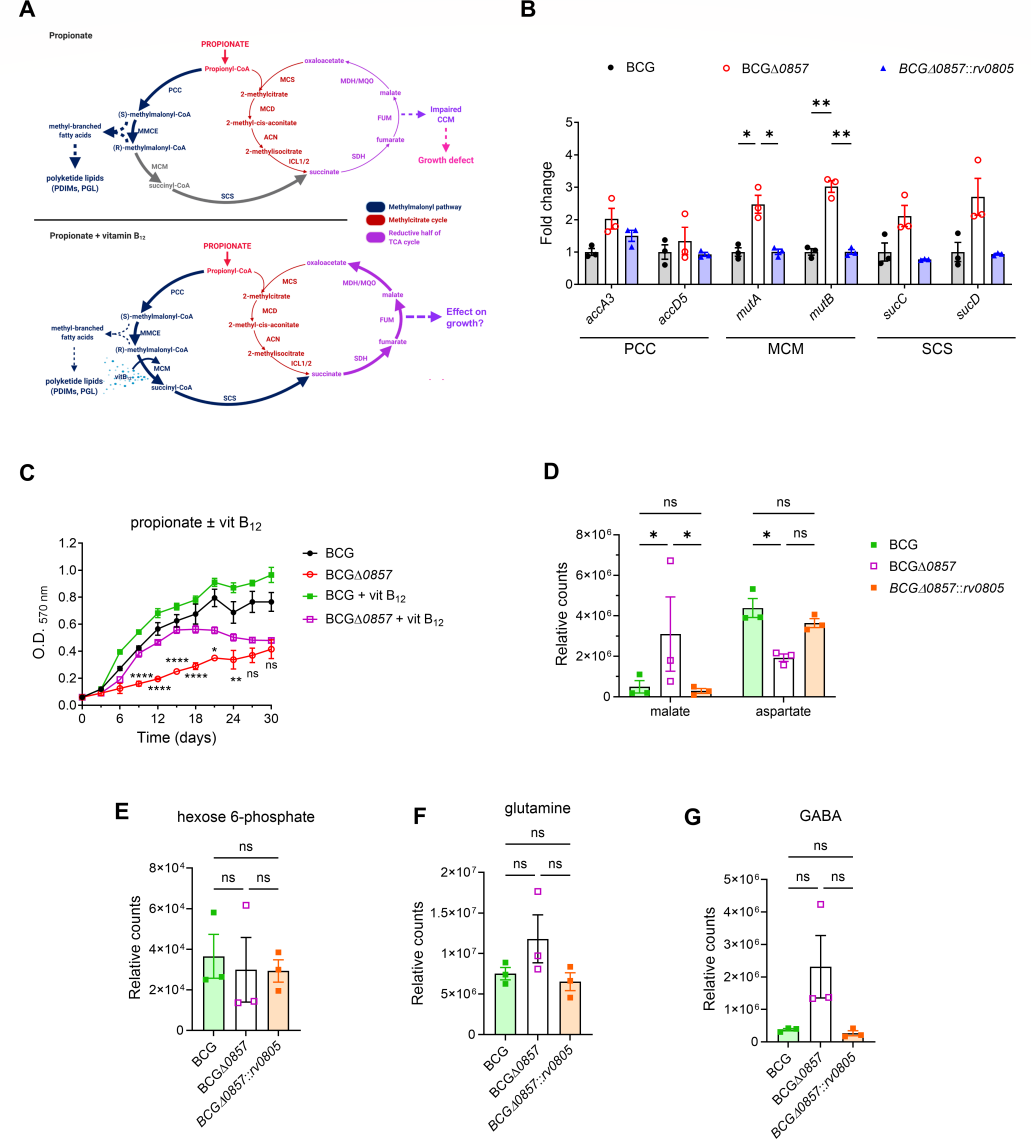
Vitamin B_12_ supplementation rescues CCM metabolite levels and growth defect in propionate. (**A**) Propionate metabolism with or without vitamin B_12_ supplementation. (**B**) QRT-PCR analysis of MM pathway genes in exponential phase cultures grown in propionate minimal medium (*n* = 3). 16s was used as the reference gene. (**C**) Growth curves in propionate minimal medium with or without 10 μg/mL vitamin (vit) B_12_ supplementation (*n* = 4). Rescued steady-state metabolite levels of (**D**) malate and aspartate, (**E**) hexose 6-phosphate, (**F**) glutamine, and (**G**) GABA in propionate media supplemented with vitamin B_12_ (*n* = 3). For B–G, results are presented as mean ± SEM from biological replicates. For B, D–G, statistical significance was determined by one-way ANOVA with Tukey’s multiple comparison test. In C, statistical significance for the growth of BCGΔ*0857* between propionate and propionate ± vit B_12_ was determined by two-way ANOVA with Tukey’s multiple comparison test. In B, asterisks are used only for comparisons where the *P*-value is less than 0.05. *P < 0.05; **P < 0.01; ****P < 0.0001; ns, not significant.

The results shown above suggested that propionate is shuttled into lipid anabolism in the BCGΔ*0857* strain. We, therefore, hypothesized that the activation of the MM pathway may shuttle propionate back into the MCC pathway via the enzymatic activity of MCM and SCS (Fig. 6A). To confirm this, we first verified the expression of key enzymes involved in the MM pathway. Genes encoding propionyl-CoA carboxylase (PCC genes *accA3* and *accD5*) and succinyl-CoA synthetase (SCS genes *sucC* and *sucD*) subunits were expressed at similar levels in all strains, whereas the expression of *mutA* and *mutB* encoding methylmalonyl-CoA mutase (MCM) subunits was higher in BCGΔ*0857* than BCG and BCGΔ*0857::rv0805* (Fig. 6B). Supplementation of the growth media with Vitamin B_12_ rescued the growth defect of BCGΔ*0857* in propionate medium (Fig. 6C), and levels of the malate (Fig. 6D) and normalized the levels of other central carbon metabolites (Fig. 6E through G). However, aspartate levels were still lower in BCGΔ*0857* than in BCG (Fig. 6D). Together, this suggests that the absence of Rv0805 results in improper carbon rationing at the propionyl-CoA node, which can be rescued on vitamin B_12_ supplementation by redirecting the carbon back from MM-CoA to CCM.

### Loss of *bcg_0857*-mediated modulation of methyl-branched lipid synthesis diminishes carbon flow toward CCM

**Fig 7:**
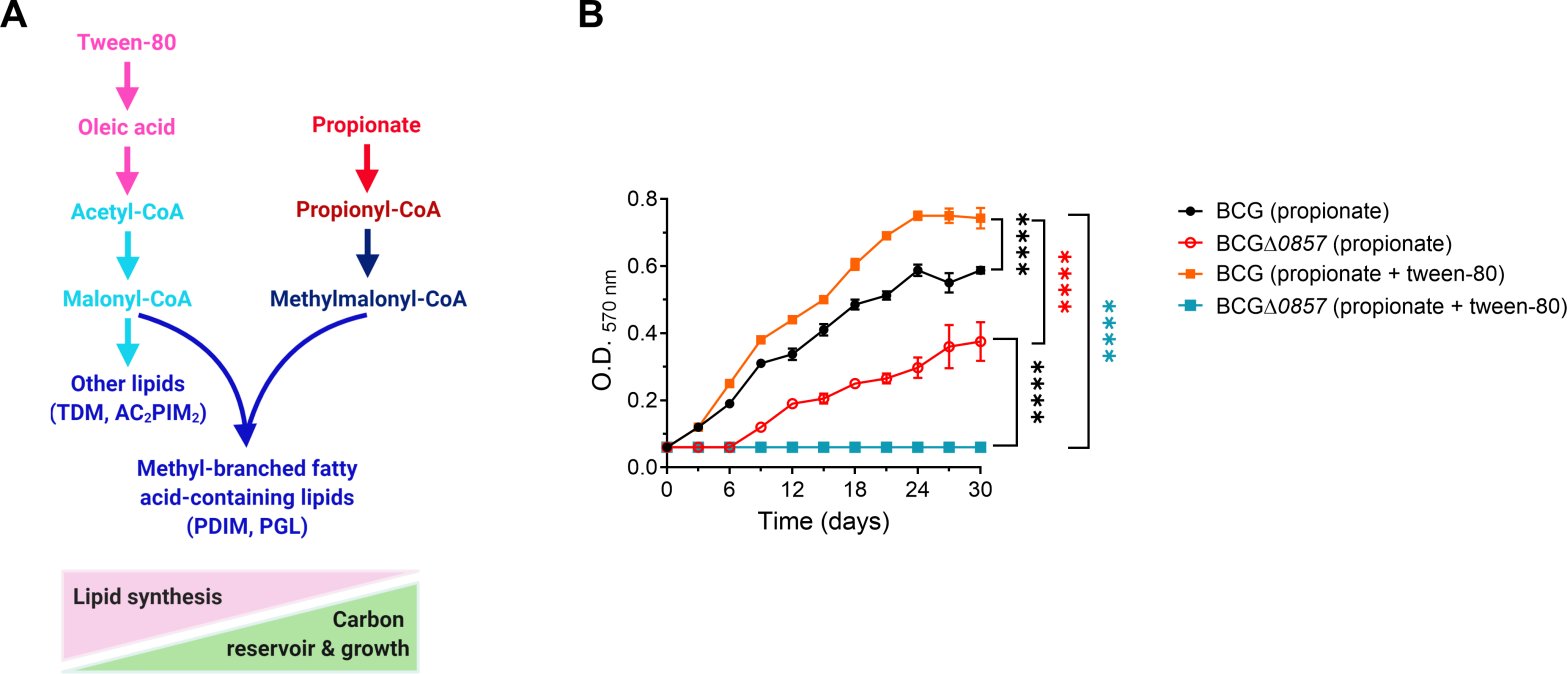
The addition of Tween-80 exacerbates the growth defect in propionate. (**A**) Probable effect of Tween-80 on the growth of BCGΔ0*857* in propionate. Tween-80 is catabolized as oleic acid, which is further converted into acetyl-CoA. Supplementation of Tween-80 in propionate-grown BCGΔ*0857* may lead to even higher synthesis of methyl-branched as well as other cell wall lipids in BCGΔ*0857*. This may replace more methylmalonyl-CoA from the propionyl-CoA node and reduce carbon flux towards CCM, adversely affecting the growth of BCGΔ*0857* on propionate. (**B**) Growth curves in propionate media supplemented with 0.05% Tween-80 (*n* = 4), using propionate minimal media as control. Results show mean ± SEM from biological replicates, and statistical significance was determined by two-way ANOVA with Tukey’s multiple comparison test. ****P < 0.0001.

A higher conversion of propionyl-CoA to (S)- and (R)-methylmalonyl-CoA could lead to increased synthesis of methyl-branched fatty acid-containing polyketide lipids, such as PDIMs and PGLs, potentially compromising CCM and impairing the growth of strains lacking Rv0805 (Fig. 6A). To test this, we sought to perturb carbon allocation further and analyze its effect on growth. We grew the BCGΔ*0857* strain in propionate-containing medium, supplemented with 0.05% Tween-80. Tween-80 is catabolized to form oleic acid, ultimately generating acetyl-CoA that can be converted to malonyl-CoA (37) (Fig. 7A). The addition of Tween-80 and consequent elevation of malonyl-CoA pools could increase the conversion of propionyl-CoA to methylmalonyl-CoA and deplete the flux of carbon toward MCC and, thus, restrict the growth of BCGΔ*0857* in propionate-containing media. As expected, BCGΔ*0857* failed to grow in propionate + Tween-80 media. In contrast, the wild-type strain exhibited higher growth (Fig. 7B). Overall, our results indicate that a balance exists between lipid synthesis and central carbon metabolism for growth, which is regulated by the catalytic activity and membrane localization of Rv0805.

## DISCUSSION

The ability of mycobacteria to thrive within the lipid-rich but nutrient-limited environment of the host is underpinned by precise coordination between carbon metabolism and cell envelope composition. In this study, we identify the cell envelope-associated metallophosphoesterase BCG_0857/Rv0805 as a factor involved in this coordination during growth on propionate, an intermediate of host cholesterol catabolism. Loss of BCG_0857 not only perturbs lipid homeostasis but also causes a metabolic bottleneck at the malate/aspartate node, highlighting how structural changes at the cell surface reverberate into central carbon metabolism. These findings define a mechanistic link between envelope architecture and carbon flux control in mycobacteria.

The phenotype of the *bcg_0857* deletion mutant parallels features observed in *M. tuberculosis* Δ*icl1/2*, one of the most attenuated strains of *Mtb* and a classic model for impaired propionate utilization (13, 18, 38, 39). Like Δ*icl1/2*, BCGΔ*0857* exhibits a pronounced growth defect in propionate and a depletion of critical CCM metabolites, accompanied by impaired membrane potential. However, unlike Δ*icl1/2*, where isocitrate lyase activity is absent and toxic MCC intermediates accumulate downstream of propionyl-CoA, BCGΔ*0857* retains ICL activity and does not accumulate these intermediates. Instead, the absence of BCG_0857 leads to an excessive diversion of propionyl-CoA into methylmalonyl-CoA and onward into the synthesis of methyl-branched polyketide lipids, resulting in elevated PDIM and PGL. This pattern is consistent with prior observations that mycobacteria couple cholesterol metabolism through excess propionyl-CoA pools to increased PDIM/PGL production and the formation of longer methyl-branched acyl chains (18, 20). In BCGΔ*0857*, this diversion of propionyl-CoA toward methyl-branched lipid synthesis appears to occur at the expense of MCC flux, resulting in reduced ^13^C-carbon incorporation into 2-methylcitrate, depletion of malate and oxaloacetate, and lowered hexose-6-phosphate steady-state levels, ultimately impairing gluconeogenesis and biomass production.

Although a modest increase in ^13^C labeling of pyruvate and transcriptional induction of *pckA*, *ppdk*, *pca*, and *icl2* was observed in BCGΔ*0857*, fractional ^13^C enrichment remained low in all strains (~1%–3%). Absolute pyruvate levels were unchanged, and carbon flux into downstream anaplerotic outputs, including phosphoenolpyruvate (PEP), was not restored in BCGΔ*0857*. These changes, therefore, reflect a compensatory remodeling of the PEP-pyruvate-oxaloacetate or anaplerotic node rather than productive rerouting of the propionate-derived carbon, reinforcing that impaired MCC flux is the primary driver of defective anaplerosis and gluconeogenesis in BCGΔ*0857*.

BCGΔ*0857* also showed reduced *prpD* and increased *icl2* expression (Fig. S4F). However, reduced ^13^C incorporation into MCC intermediates and depletion of malate and oxaloacetate indicate that this altered gene expression was insufficient to restore flux, and impaired upstream carbon partitioning was the primary defect.

Proper gluconeogenesis is essential for infection, intracellular survival, and the biosynthesis of vitamins, lipids, nucleotides, reducing equivalents, and structural components needed for cell division (40). In BCGΔ*0857*, reduced propionate assimilation not only limits these anabolic processes but also compromises membrane potential—an equally critical determinant of active replication and long-term viability (41). The excess accumulation of PDIM and PGL in BCGΔ*0857* may also have consequences for host–pathogen interactions (42–44). PDIM has been shown to spread into host epithelial membranes and promote infectivity (45). Thus, elevated PDIM levels in BCGΔ*0857* could translate into altered infectivity *in vivo*, highlighting another way in which Rv0805-mediated carbon partitioning may shape both bacterial physiology and host interactions. Phosphatidylmyo-inositol Mannosides (PIMs) are key regulators of mycobacterial envelope integrity and permeability. Disruption of PIM biosynthesis alters the uptake of lipophilic compounds, such as norfloxacin and chenodeoxycholate (46, 47). Thus, the increased Ac_2_PIM_2_ levels in BCGΔ*0857* likely contribute to its altered permeability to lipophilic agents.

An example of another PDE influencing cell wall physiology is Rv1339, whose overexpression in *M. smegmatis* lowers cAMP, perturbs peptidoglycan precursor pools, and compromises envelope integrity (48). Although the mechanistic link to cAMP signaling remains correlative, this underscores how cyclic-nucleotide-modulating enzymes can broadly affect cell envelope homeostasis. Given the altered envelope composition of the BCGΔ*0857* strain and the requirement for Rv0805 membrane localization for optimal growth, Rv0805 may likewise regulate envelope-associated processes beyond cAMP hydrolysis. Indeed, despite previous reports linking Rv0805 to altered cAMP turnover (30), we did not detect significant changes in either extracellular or intracellular cAMP levels between the wild type and BCGΔ*0857* in propionate. These findings suggest that Rv0805 acts through an alternative or membrane-proximal substrate, integrating its catalytic and membrane localization-dependent functions to coordinate envelope lipid balance with metabolic regulation during propionate utilization.

Notably, BCGΔ*0857* also displayed a growth defect on glycerol, suggesting Rv0805 regulates carbon partitioning beyond propionate metabolism. During growth on glycerol and Tween-80, mycobacteria can operate a reverse MCC to supply propionyl-CoA for virulence lipid synthesis (14). In the absence of BCG_0857, this reverse routing may be enhanced, diverting more glycerol-derived carbon into methyl-branched lipid synthesis at the expense of CCM and biomass production.

The growth defect of BCGΔ*0857* in propionate was further exacerbated when supplemented with Tween-80 (Fig. 7A). Tween-80 supplementation increases acetyl-CoA availability through fatty acid metabolism, which in the context of BCGΔ*0857* likely intensifies the diversion of propionyl-CoA and methylmalonyl-CoA toward lipid biosynthesis at the expense of central carbon flux, thereby halting growth (Fig. 7A). This contrasts with the *Mtb Δicl1* mutant, where acetate supplementation alleviates propionate toxicity by promoting lipid synthesis and thereby reducing accumulation of toxic MCC intermediates (20). In BCGΔ*0857*, where ICL activity is retained, growth impairment arises from carbon diversion into lipids rather than MCC toxicity. Consistent with our findings, mutations in the *pta-ackA* operon, that limit acetyl-CoA production and deplete acetyl-CoA pools, alleviate the propionate growth defect of BCGΔRv0805 (30). Together, these findings underscore the tight control of propionyl-CoA partitioning and reveal Rv0805 as a key regulator that prevents excessive lipid commitment, which can compromise metabolic fitness.

Vitamin B_12_ supplementation rescues the propionate growth defect in both Δ*icl1/2* (4) and BCGΔ*0857*, but via distinct routes. In Δ*icl1/2*, vitamin B_12_-driven activation of the MM pathway bypasses the MCC block, reducing accumulation of toxic 2-methylcitrate/2-methylisocitrate and replenishing CCM intermediates. In BCGΔ*0857*, where MCC flux is reduced rather than blocked, vitamin B_12_ enables methylmalonyl-CoA mutase (MCM) to convert methylmalonyl-CoA into succinyl-CoA, shunting carbon back into the CCM (Fig. 6A). This effect is reinforced by higher *mutAB* and *sucCD* (encoding MCM and SCS subunits) expression in BCGΔ*0857*, which likely supports the observed recovery of malate and other metabolites. While aspartate (proxy for oxaloacetate) levels remain low after vitamin B_12_ supplementation, restoring malate and downstream gluconeogenic intermediates is sufficient to reestablish growth. Oxaloacetate is rapidly consumed in gluconeogenesis, transamination, and condensation reactions (6), so its steady-state levels may remain low despite restored anaplerotic flux. Supporting this, elevated *pca*, *pckA*, and *ppdk* expression in BCGΔ*0857* suggests increased turnover rather than accumulation (Fig. 5F). Thus, vitamin B_12_ restores effective carbon flux without fully normalizing oxaloacetate-derived pools.

The ability of *Mtb rv0805* to restore growth and metabolic phenotypes in BCGΔ*0857* demonstrates functional conservation of Rv0805 across MTBC species, supporting the use of BCG as a valid and informative model for dissecting its metabolic role. However, while this study suggests that Rv0805-mediated regulation of carbon partitioning may influence host-pathogen interactions, it is important to note that these experiments were conducted in BCG, which is attenuated due to loss of several virulence-associated regions, including ESX-1 (31–33). Therefore, direct extrapolation to *Mtb* pathogenesis should be made with caution, and future studies using *Mtb* infection models are required to determine how Rv0805-dependent control of propionyl-CoA flux and cell envelope composition impacts virulence, persistence, and immune modulation *in vivo*.

Our findings, therefore, suggest that BCG_0857/Rv0805 is involved in both cell envelope lipid homeostasis and carbon routing at the propionyl-CoA node. The parallels with the highly attenuated Δ*icl1/2* strain further support a role for Rv0805 in intracellular survival (30) and perhaps persistence, a hypothesis that warrants direct *in vivo* testing. Beyond its contribution to propionate utilization, BCG_0857 may act as a general integrator of envelope architecture and metabolic adaptability. The fact that vitamin B_12_ supplementation can bypass the BCGΔ*0857* defect underscores the metabolic plasticity of mycobacteria. It raises the possibility of targeting propionyl-CoA partitioning or cofactor availability to disrupt this balance. Such interventions could represent novel strategies to weaken the metabolic and structural robustness that underlie mycobacterial pathogenesis.

## MATERIALS AND METHODS

### Mycobacterial strains and culture conditions

A list of all strains used in this study is provided in Table 1. BCG was cultured in OADC (oleic acid-albumin-dextrose-catalase) agar or OADC-glycerol media (Table 2) under static conditions at 37°C in a humidified incubator located in the bio-safety level 2 (BSL2) facility. Hygromycin B and kanamycin sulfate were included at a final concentration of 50 μg/mL and 10 μg/mL, respectively, wherever necessary.

**TABLE 1:** List of strains used in this study

Strain	Description	Reference
BCG/BCG wild type	*M. bovis* BCG str. Pasteur 1173P2	This study
BCGΔ*0857*	*M. bovis* BCG deleted for the *bcg_0857* gene (600 bp of *bcg_0857* intragenic region deleted)	
BCGΔ*0857*::*rv0805*	BCGΔ*0857* expressing *rv0805* under *rv0805* promoter at the L5 *att* site with the help of an integrated plasmid pMV306 (Kan^+^)	
BCGΔ*0857*::*rv0805_N97A_*	BCGΔ*0857* expressing *rv0805*_N97A_ under *rv0805* promoter at the L5 *att* site with the help of pMV306 (Kan^+^)	
BCGΔ*0857*::*rv0805_Δ40_*	BCGΔ*0857* expressing *rv0805_Δ40_* under *rv0805* promoter at the L5 *att* site with the help of pMV306 (Kan+)	
BCG_WT_ *bcg_0857 prom_luxAB*	*M. bovis* BCG expressing *luxAB* reporter gene under *rv0805* promoter from a multi-copy plasmid pMV1025 (Hyg^+^)	
BCG_WT_*noprom_luxAB*	*M. bovis* BCG harboring *luxAB* reporter gene with *no* promoter in pMV1025 (Hyg^+^)	

**TABLE 2:** List of growth media used in this study

Medium	Composition
OADC	7H9 base medium supplemented with 10% OADC (oleic acid-albumin-dextrose-catalase), 0.2% glycerol, and 0.05% Tween-80
No carbon	7H9 base medium supplemented with 0.025% Tyloxapol
Cholesterol	7H9 base media supplemented with 250 μM cholesterol and 0.125% Tyloxapol as a vehicle of cholesterol
Glycerol	7H9 base medium supplemented with 0.2% glycerol and 0.025% Tyloxapol
Propionate	7H9 base medium supplemented with 0.2% sodium propionate and 0.025% Tyloxapol
OADC agar	7H10 agar supplemented with 0.5% glycerol and 10% OADC (oleic acid-albumin-dextrose-catalase)
Cholesterol agar	7H10 agar supplemented with 250 μM cholesterol and 0.125% Tyloxapol as a vehicle of cholesterol

### Generation of BCGΔ*0857* and complement strains

Upstream and downstream flanking regions of *bcg_0857* were amplified using BCG genomic DNA with primers listed in Table 3 and cloned separately into pBKS, generating pBKS-*bcg_0857* upstream and pBKS*-bcg_0857* downstream. The upstream and downstream flanking portions of bcg_0857 were then subcloned into p2NIL, generating p2NIL-*bcg*Δ*0857*. A marker cassette (~8 kb) was then cloned into this (by *PacI* digestion) to generate p2NIL-*bcg*Δ*0857*-Pac.

**TABLE 3:** List of other primers used in this study

Primer name	5' to 3' sequence	Purpose
*rv0805/bcg_0857* Nterm RT FWD	CGGCCGGATTACGTTCTCTT	Screening of BCG and BCGΔ*0857*
*bcg_0857* Cterm RT REV	TCAGTCGACGGGACTTCGC	
BCG *attB* FWD	GATGTCTGTCACCACGTACAGTCGC	Screening of BCGΔ*0857*::*rv0805/rv0805_N97A_*
*bcg_0857* upstream FWD	TCGATCAAGCTTCGGACGCGG	Amplification of flanking region of ~1 kbp at the 5′ end of *bcg_0857*
*bcg_0857* upstream REV	ATTGGTTCTAGAGTTCGAGCAGTTCGCCC	
*bcg_0857* downstream FWD	ACCGTCGCTTCTAGAGGAACGCGTG	Amplification of flanking region of ~1 kbp at the 3′ end of *bcg_0857*
*bcg_0857* downstream REV	GATTCAACGGATCCGCCATGGTTGGCCG	

BCG was electroporated with p2NIL-*bcg*Δ*0857*-Pac and, after recovery, plated onto OADC agar containing cycloheximide (100 μg/mL), hygromycin, kanamycin, and X-gal (50 μg/mL). Following 4–5 weeks of incubation, blue colonies [putative “single-cross overs” (SCOs)] were confirmed by colony PCR using the primers *bcg_0857*fwd and *bcg_0857*downstreamrev (Table 3). SCOs were then streaked out onto OADC-glycerol agar (no antibiotics) to allow a second recombination event, generating either wild type or deleted *bcg_0857* locus. Next, a scoopful of SCOs was plated onto OADC-glycerol agar supplemented with 2% sucrose. Only bacteria that have undergone a second crossover event would grow on sucrose-containing media. Colonies were screened for the deletion of the *bcg_0857* gene by colony PCR using primers *bcg_0857*fwd and *bcg_0857*downstreamrev (Table 3). Complement strains were generated by introducing the *rv0805* or *rv0805_N97A_* gene, along with an upstream ~150 bp intergenic region (available in the laboratory), to serve as an endogenous promoter at the L5 *att* locus in the BCGΔ*0857* genome (Fig. S1A). BCGΔ*0857* was electroporated with pMV306-*rv0805prom-rv0805/rv0805_N97A_*/*rv0805_Δ40_* and, after recovery, plated onto OADC agar containing kanamycin. Individual transformants were confirmed by PCR using BCG *attB* FWD and *rv0805/bcg_0857* Nterm RT FWD, which yielded the expected 1.5 kbp PCR amplicon.

### Growth analysis, spot assay, and susceptibility to propionate

For all experiments, BCG was grown in OADC-glycerol medium until the mid-exponential phase. For growth analysis, cells were inoculated in either glycerol or propionate minimal media (Table 2) in biological and experimental duplicates, to a starting optical density at 570 nm (O.D.) of 0.05, and incubated at 37 °C under static conditions. Culture was resuspended at specific time points, and bacterial growth was assessed by measuring optical density (O.D.) at 570 nm.

For spot assay, 200 µL O.D._570 nm_ = 1 were washed twice in assay buffer (phosphate-buffered saline [PBS] supplemented with 0.025% Tyloxapol), resuspended in the same buffer, and 5 µL spotted on cholesterol, OADC, or OADC agar supplemented with 10 µg/mL malachite green, 10 µg/mL crystal violet, or 0.005% SDS. The plates were incubated at 37°C for 16–25 days and imaged.

To monitor the effect of propionate toxicity, cells were inoculated in glycerol minimal media (Table 2) with increasing concentrations of sodium propionate. Cultures were seeded with a starting O.D. at 570 nm of 0.05 and incubated at 37°C under static conditions until the control culture (glycerol alone) reached the mid-exponential phase. Bacterial growth (O.D._570 nm_) was measured, and relative growth compared to propionate-free media (glycerol alone) was determined.

To monitor susceptibility to ionophores and antibiotics, cells were inoculated in glycerol medium containing varying concentrations of a specific antibiotic and incubated at 37°C under static conditions until the control culture (glycerol alone) reached the mid-exponential phase. Bacterial growth (O.D._570 nm_) was measured, and relative growth compared to ionophore or antibiotic-free media (glycerol alone) was estimated.

### Preparation of mycobacterial cell lysate and Western blot analysis

For preparation of cell lysates, cells were harvested, washed with TBST buffer (10 mM Tris-Cl (pH 8.2), 0.9% NaCl, and 0.1% Tween-80), and lysed by bead beating against 0.5 mm diameter glass beads (BioSpec Products, USA) in buffer containing 50 mM Tris-Cl (pH 8.2), 100 mM NaCl, 10% glycerol, 5 mM β-mercaptoethanol (β-ME), 2 mM phenylmethyl sulfonyl fluoride (PMSF), and 1 mM benzamidine hydrochloride. The protein concentration of the lysates was estimated using the Bradford method.

Protein samples were electrophoresed on SDS (sodium dodecyl sulfate) PAGE polyacrylamide gel (12% acrylamide, 1.2% bis-acrylamide) and transferred to PVDF (polyvinylidene fluoride) membranes (Millipore, USA) in transfer buffer (25 mM Tris, 192 mM glycine, 20% methanol, pH 8.3) for 2 h at 200 mA. After transfer, the membrane was incubated in blocking agent for 1 h at 25°C, followed by incubation with primary antibody overnight at 4°C with gentle agitation. Rv0805-specific monoclonal antibody was used at a 1:50 dilution. The anti-CRP polyclonal antiserum was used at a dilution of 1:5,000. Next, membranes were washed thrice with TBST and incubated with anti-mouse IgG conjugated to horse-radish peroxidase (1:5,000) or anti-rabbit IgG conjugated to horse-radish peroxidase (1:25,000) for 1 h at 25°C. The membranes were washed three times with TBST, and the bound antibody was detected using Enhanced Chemiluminescence substrate (ECL Plus, GE Healthcare, UK).

### Promoter activity and growth analysis

*The bcg_0857* promoter (150 bp upstream region of *bcg_0857*) was cloned upstream of the luciferase reporter gene (*luxAB*) in the pMV1025 vector, and the BCG wild-type strain was electroporated with this construct. A control strain harboring pMV1025-*no promoter-luxAB* was also generated as a control to ascertain no expression of *luxAB* in the absence of any promoter (Table 1). BCG_WT_*bcg_0857 prom_luxAB* strain was grown in glycerol or propionate minimal media, and *bcg_0857* promoter activity was monitored at specific time-points along with growth. For promoter activity analysis, 20 μL of the resuspended culture was diluted in 90 μL of TBST on a Luminance white plate, and 10 μL of 1% decanal (Sigma), prepared in 70% ethanol, was added. Luciferase activity was measured immediately using an Infinite M200 PRO (Tecan, Switzerland) luminometer and normalized to the growth rate.

**TABLE 4:** List of primers used for qRT-PCR

Primer name	5' to 3' sequence	Gene	BCG ortholog
16S RT FWD	CGAGCGTTGTCCGGAATTA	*16s*	*16s*
16S RT REV	TCCACCGCTACACCAGGAAT		
accA3 RT FWD	ACATCACCGAGGATCCCACCC	*rv3285*	*bcg_3314*
accA3 RT REV	GGGTGGAACTTTGTCACCGGC		
accD5 RT FWD	CACCATGGAAGAACTCGGCGG	*rv3280*	*bcg_3309*
accD5 RT REV	GCGTCGGTGGAGTTGTTGGG		
bcg_1605 RT FWD	AAAGATACTGGTGGGCGAGGC	*rv1554*	*bcg_1605*
bcg_1605 RT REV	CGTTCAGCACTACGACAACCG		
frdA RT FWD	TTCGTACGTGAGCTGTGCCG	*rv1552*	*bcg_1604*
frdA RT REV	GGCATATAGCCCGGGAAGCG		
frdD RT FWD	GTACTGGCCCTGTTCCATGC	*rv1555*	*bcg_1606*
frdD RT REV	CATGCCGTAACACCACAGGG		
fum RT FWD	GTCGTCCAACGACACCTTCCC	*rv1098c*	*bcg_1158c*
fum RT REV	CGACTTCACCACCGTGTGCC		
icl1 RT FWD	TGTGGCTGATGTTCCCACGG	*rv0467*	*bcg_0507*
icl1 RT REV	GGTGCGGTAGAAGCCTTCCC		
icl2 RT FWD	GCATTTTCGGCTTGGTCTCCG	*rv1915/ rv1916*	*bcg_1954*
icl2 RT REV	TCTCCGTAGGTCATCAGGCCC		
mdh RT FWD	CGAAGTGGTCAACGACCAGGC	*rv1240*	*bcg_1300*
mdh RT REV	TCGAGACCCAATCGTCCGCC		
mutA RT FWD	TTCGACGTGGCGATTCCCG	*rv1492*	*bcg_1555*
mutA RT REV	CTTCGACGAACCACGACCCG		
mutB RT FWD	CGTTCTTCTGGGGCATCGGG	*rv1493*	*bcg_1556*
mutB RT REV	GCAGCGAAAGGGATTTGGCG		
mqo RT FWD	CCTCGCCAAACAACTCATCGG	*rv2852c*	*bcg_2872c*
mqo RT REV	TCAACTTGCGCTTTTCGCCG		
mez RT FWD	GCGGTGTCTCTGGATGTCGG	*rv2332*	*bcg_2354*
mez RT REV	GCGGAAATAACCGTTGAGCCG		
pca_RT FWD	GCAAGAAGCAACAGCCACCG	*rv2967c*	*bcg_2988c*
pca_RT REV	ACCCGGATCGGTAAGGTCGC		
pckA_RT FWD	CTCACTGCGTATCGCGTCGG	*rv0211*	*bcg_0248*
pckA_RT REV	AGGTTGGTCTTGCCACACGC		
ppdk_RT FWD	TACCGACGACCCGTATGCCC	*rv1127c*	*bcg1188c*
ppdk_RT REV	AGGTTGCCGAATACCATCGCC		
prpC RT FWD	TCAAGGGGCGGCTACACGG	*rv1131*	*bcg_1192*
prpC RT REV	TACACCCGATGCCCGAAGCC		
prpD RT FWD	TGATCCGCGGTCTGGTAACCG	*rv1130*	*bcg_1191*
prpD RT REV	TGGTCTCTTGGTCGAGCCGC		
prpR RT FWD	TTACCGAAAGCGGGATGCGG	*rv1129c*	*bcg_1190c*
prpR RT REV	GAAGGACCTTGGTGTCGGGG		
rv0247c RT FWD	CCAGCGAGTACCGAATGGCG	*rv0247c*	*bcg_0285c*
rv0247c RT REV	ATTCGCATCAGGAAGCGGGG		
rv0248c RT FWD	GCAGGCAATGGAAGTCGGGC	*rv0248c*	*bcg_0286c*
rv0248c RT REV	ATCGGACAGCGAATTGCCGC		
rv0249c RT FWD	CATCTCGGCGTCTGGTTCGG	*rv0249c*	*bcg_0287c*
rv0249c RT REV	ACCGGTAGTAGGCCTTGCGG		
sdhA RT FWD	TGTCTTTCACGCCAAGGCCG	*rv3318*	*bcg_3384*
sdhA RT REV	CCCTTGCGGAACACGATGCC		
sdhB RT FWD	CAAGGTGCTGATGCGTGACC	*rv3319*	*bcg_3385*
sdhB RT REV	CGCTGGTGATCAGGTACGGT		
sdhC RT FWD	GCTGGCGACCTACAAGACCC	*rv3316*	*bcg_3382*
sdhC RT REV	GATGATCCACAACATCAGCCGC		
sdhD RT FWD	TTCGCCTGGCTGTTCATGCG	*rv3317*	*bcg_3383*
sdhD RT REV	TTGAAGTCCAGGCGATACACGC		
sucC RT FWD	AGGGCGTTGACCTAGATTTCGC	*rv0951*	*bcg_1005*
sucC RT REV	ATCTTGTGGTCAGGCGTCCG		
sucD RT FWD	TGGTCTGGTGTCCAAGTCGGG	*rv0952*	*bcg_1006*
sucD RT REV	TGTGGGTAGTGCCAATCACCG		
rv0805 RT FWD	GCTGAAGAGTTGGCCACGCC	*rv0805*	*bcg_0857*
rv0805 RT REV	TCGAGTAGTGCAGGTGCCCG		

### Radioimmunoassay

Iodinated 2′-O-monosuccinyl adenosine-3′,5′-cyclic monophosphate tyrosyl methyl ester (2′-O-MS-cAMP-TME) (BRIT, India) was used for radioimmunoassay. Radioimmunoassay was carried out by setting up a competition between iodinated 2′-O-MS-cAMP-TME and unlabeled cAMP. The amount of cAMP-specific antiserum used was standardized so that 30%–50% of input iodinated 2′-O-MS-cAMP-TME (input ~25,000 cpm) bound to the antiserum in the absence of competing cAMP. The assay was set up in 50 mM sodium acetate buffer (pH 4.75) containing 5 mg/mL BSA in a total volume of 300 μL and allowed to equilibrate by overnight incubation at 4°C. Free cAMP was separated from antibody-bound cAMP by the addition of 1 mL activated charcoal (2 mg/mL) and BSA (1 mg/mL) in 50 mM potassium phosphate buffer, pH 6.3. Charcoal was then recovered by centrifugation at 4,000 × *g* for 20 min at 4°C. The supernatant was discarded, and the radioactivity associated with the charcoal pellets was measured using a γ-counter (PerkinElmer, USA). A standard curve, consisting of 12–15 known concentrations of cAMP, was used to calculate the concentrations of cAMP in the unknown samples.

For sample preparation, samples were diluted to appropriate amounts in 50 mM Na-acetate buffer (pH 4.75) and acetylated to increase the sensitivity of the radioimmunoassay. For acetylation, the samples were mixed with 0.02 volume of triethylamine by vortexing. This was followed by the rapid addition of 0.01 volume of acetic anhydride just below the surface of the sample solution and vortexing. The cAMP reference standard was acetylated in a similar manner. Appropriate amounts were used for radioimmunoassay.

For the preparation of intracellular cAMP measurement, approximately 200 µL of O.D. 1 equivalent culture was centrifuged and resuspended in a 200 µL volume of 0.1 N HCl. The sample was then boiled at 95°C for 10 min and stored at −70°C until measurement. For extracellular cAMP measurement, 0.1 volume (50 µL) of 1 N HCl was added to 450 µL of culture supernatant, boiled at 95°C for 10 min, and then stored at −70°C until measurement.

### RNA isolation, cDNA preparation, and gene expression analysis by qRT-PCR

Mid-exponential-phase BCG cultures were centrifuged, resuspended in TRI Reagent (RNAiso Plus, TaKaRa, Japan), and lysed using 0.5-mm-diameter glass beads (BioSpec Products, USA). The lysate was then heated at 65°C for 5 min and centrifuged at 16,000 × *g* for 10 min at 4°C. The supernatant was collected and mixed with chloroform, followed by centrifugation at 16,000 × *g* for 15 min at 4°C. The upper aqueous phase was collected, and RNA was precipitated with isopropyl alcohol. The RNA pellet was washed with 75% ethanol, dissolved in RNase-free Milli-Q water, and treated with RNase-free DNase (Thermo Scientific). Two micrograms of RNA was used for reverse transcription using 200 units of reverse transcriptase (Thermo Scientific, USA). Sequences of primers used to study the transcript level of different genes are shown in Table 4. Real-time qRTPCR was performed using SYBR Premix *Ex Taq* (Tli RNase H Plus) on a CFX96 Touch real-time PCR detection system (Bio-Rad, USA). The housekeeping gene 16S rRNA was used as an internal control for normalization.

### Metabolite extraction, liquid chromatography-mass spectrometry

Strains were cultured on OADC-glycerol agar. BCG was initially grown in 7H9 media supplemented with 0.2% glucose, 0.5% fraction V BSA (HiMedia, India), 0.085% NaCl, and 0.05% Tyloxapol until the late exponential phase and then inoculated onto 22 mm diameter, 0.22 μm pore size nitrocellulose filters under vacuum filtration. Laden filters were then placed on top of chemically equivalent agar media (described above) and allowed to grow at 37°C for 5 days. Filters were then transferred for 24 h into 7H10 plates supplemented with 0.5% fraction V BSA, 0.05% Tyloxapol, 0.085% NaCl, containing 0.2% propionate, 0.2% propionate supplemented with 10 μg/mL of vitamin B_12_, or 0.2% [U-^13^C]-propionate. Bacteria were metabolically quenched by plunging cell-laden filters into acetonitrile/methanol/H_2_O (2:2:1, by vol.), pre-cooled to −40°C, and metabolites were extracted by mechanical lysing of the entire bacteria-containing solution with 0.1 mm acid-washed Zirconia beads (Sigma). Lysates were clarified by centrifugation and then filtered through 0.22 μm Spin-X column filters (Costar). The bacterial biomass of individual samples was determined by measuring the residual protein content of the metabolite extracts using the BCA Protein Assay Kit (Thermo Fisher Scientific).

Aqueous normal-phase liquid chromatography of the metabolites was performed using an Agilent 1290 Infinity II LC system equipped with a binary pump, temperature-controlled auto-sampler (set at 4°C), and temperature-controlled column compartment (set at 25°C), containing a Cogent Diamond Hydride Type C silica column (150 mm × 2.1 mm; dead volume 315 µL). A flow rate of 0.4 mL/min was used. Elution of polar metabolites was carried out using solvent A, consisting of deionized water (Resistivity ~18 MΩ cm), 0.2% acetic acid, and solvent B, composed of 0.2% acetic acid in acetonitrile. The following gradient was used: 0 min 85% B; 0–2 min 85% B; 3–5 min to 80% B; 6–7 min 75% B; 8–9 min 70% B; 10–11 min 50% B; 11.1–14 min 20% B; 14.1–25 min hold 20% B followed by a 5 min re-equilibration period at 85% B at a flow rate of 0.4 mL/min. Accurate mass spectrometry was carried out using an Agilent Accurate Mass 6545 QTOF apparatus. Dynamic mass axis calibration was achieved by continuous infusion, post-chromatography, of a reference mass solution using an isocratic pump connected to an ESI ionization source, operated in the positive-ion mode. Nozzle voltage and fragmentor voltages were set at 2,000 V and 100 V, respectively. The nebulizer pressure was set at 50 psig, and the nitrogen drying gas flow rate was set at 5 L/min. The drying gas temperature was maintained at 300°C. The MS acquisition rate was 1.5 spectra/s, and *m*/*z* data ranging from 50 to 1,200 were stored. This instrument enabled accurate mass spectral measurements with an error of less than 5 parts-per-million (ppm), a mass resolution ranging from 10,000 to 25,000 over the *m*/*z* range of 121–955 atomic mass units, and a 100,000-fold dynamic range with picomolar sensitivity. The data were collected in the centroid 4 GHz (extended dynamic range) mode. Detected *m*/*z* ratios were deemed to be identified metabolites based on unique accurate mass-retention time identifiers for masses exhibiting the expected distribution of accompanying isotopomers. The typical variation in abundance for most metabolites stayed between 5% and 10% under these experimental conditions. All non-labeled metabolomics data were analyzed using Agilent MassHunter Qualitative Analysis B.07.00. Targeted labeled metabolic data were performed using targeted features extractions using Profinder 8.0 (Agilent Technologies, USA), exported, and analyzed via Agilent VistaFlux software for data visualization.

As 2-methylcitrate and 2-methylisocitrate are structural isomers with identical exact masses and similar chromatographic behavior, the LC-MS platform used in this study cannot distinguish them without targeted analytical standards or MS/MS fragmentation (49). Accordingly, the measured signal represents the collective pool of methylcitrate and methylisocitrate (methyl[iso]citrate), which we refer to as methylcitrate for simplicity of presentation.

### Measurement of membrane potential

Propionate-grown and mid-exponential phase BCG cultures were centrifuged and immediately resuspended in 7H9 base media supplemented with 0.2% glycerol, 0.2% glucose, 0.085% NaCl, and 0.025% Tyloxapol. Two hundred and fifty microliters of resuspended cells was incubated with 15 µM of the fluorescent membrane-permeable dye 3,3′-diethyloxicarbocyanine chloride (DiOC_2_, Sigma) for 20 min at 25 °C in the dark. Cells were washed with 1 mL of sterile 1× PBS and resuspended in 250 μL of sterile 7H9 base medium. One hundred microliters in duplicates was aliquoted on a Costar black 96-well flat-bottom plate (Costar), and fluorescence was monitored by following excitation/emission at green 488/530 nm and red 488/610 nm using an Infinite M200 PRO (Tecan, Switzerland). The membrane potential was calculated as a ratio of red to green fluorescence. The positive control for membrane depolarization was achieved by incubating cells with 5 μM ionophore: carbonyl cyanide 3-chlorophenylhydrazone (CCCP, Sigma) along with the dye in the reaction.

### Lipid extraction

[1,2-^14^C] sodium acetate (specific activity ~37.70 mCi/mmol; BRIT, India) was added to propionate-grown exponential-phase BCG cultures at a concentration of 1 µCi/mL and incubated for an additional 7 days. The cells were then centrifuged, and the cell pellets were dried at 65°C in glass tubes. To the dried biomass, 2 mL of methanol/0.3% NaCl (10:1, vol/vol) was added, and the mixture was vortexed and sonicated at 25°C for 45 min. Two milliliters of petroleum ether was added, and the tubes were vortexed and mixed on a rotator for 20 min. The suspension was centrifuged at 3,000 rpm for 5 min, and the upper layer was collected. The extraction was repeated, and the petroleum ether fractions were pooled. The tubes were dried at 65°C, and the dried residue represented the apolar lipids.

To extract the polar lipid fraction, the cell residue was treated with 2.3 mL of chloroform/methanol/0.3% NaCl (9:10:3, by volume) and mixed on a rotator for 60 min. The sample was centrifuged for 5 min at 3,000 rpm, and the supernatant was collected. The cell pellet was again treated with 750 µL chloroform/methanol/0.3% NaCl (5:10:4, by volume), mixed on a rotator for 30 min, and then centrifuged for 3 min at 3,000 rpm. The supernatants were pooled with the supernatant from the first extraction. This was repeated once more, and 1.3 mL of chloroform and 1.3 mL of 0.3% NaCl were added to the pooled extracts, allowing two phases to form. After mixing for 5 min, the sample was centrifuged for 5 min at 3,000 rpm, and the lower layer containing polar lipids was collected using a long Pasteur pipette, transferred to a separate tube and dried at 65°C. The dried residue represented the polar lipids.

Dried polar and apolar lipids were resuspended in 200 µL chloroform: methanol (2:1, vol/vol), and equal radioactivity (~25,000 dpm) from each sample was analyzed by one- or two-dimensional thin-layer chromatography (2D-TLC). Samples were applied to silica gel 60 F_254_ plates (Merck) and resolved using solvent system A, B, or D in the case of specific apolar lipids and solvent system E for polar lipids (Table 5). Radioactivity was monitored using a phosphorimager (Bass 1800; Fuji). The intensity of spots or bands was monitored by densitometric analysis using ImageJ.

**TABLE 5:** List of TLC solvent systems used in this study

Solvent system	Run direction	Components
A	1	Petroleum ether:ethyl acetate = 96:4 (*3)
	2	Petroleum ether:acetone = 80:20
B	1	Petroleum ether:ethyl acetate = 85:15 (*3)
	2	Toluene:acetone = 85:15
D	1	Chloroform:methanol:water = 100:20:2
	2	Chloroform:acetone:methanol:water = 50:60:2.5:3
E	1	Chloroform:methanol:water = 60:30:6
	2	Chloroform:acetic acid:methanol:water = 40:25:3:6

### Intrabacterial pH measurement

pUV15-pHGFP (Addgene; plasmid #70,045), an episomal plasmid containing a hygromycin-resistant cassette and encoding a pH-sensitive ratiometric GFP [pHGFP] under the mycobacterial promoter Psymc, was used to electroporate BCG strains. BCG harboring pUV15-pHGFP was grown in glycerol + hygromycin till they reached the mid-exponential phase. Cells were centrifuged and resuspended in phosphate-citrate buffer at pH 7.4 or pH 4.4, supplemented with 0.025% Tyloxapol, to an O.D. at 570 nm of 0.5. Cells were incubated in biological replicates in 24-well plates (JetBiofil, China) at 37°C for 15 min or 8 h. Following incubation, cells were concentrated fivefold by centrifugation to increase the GFP signal. One hundred microliters of aliquots was analyzed in Infinite M200 PRO reader (Tecan, Switzerland) (Excitation = 395 & 475 nm, emission = 510 nm). Interbacterial pH was determined by interpolating the ratios of (Ex_395 nm_, Em_510 nm_) and (Ex_475 nm_, Em_510 nm_) on a standard curve.

Standard curves were generated by placing 70 µg lysates (at a concentration of 14 µg/mL) prepared from glycerol-grown BCG electroporated with pUV15-pHGFP in 100 µL of a phosphate citrate buffer series at pH 5.6 to pH 8.0 in 0.4 pH increments. The 395/475 fluorescence ratios were fitted to pH with the sigmoidal-Hill equation using Prism software (GraphPad Prism 10.6.0).

## SUPPLEMENTAL MATERIAL

Supplemental figures jb.00571-25-s0001.pdf Figures S1 to S5.

ASM does not own the copyrights to Supplemental Material that may be linked to, or accessed through, an article. The authors have granted ASM a non-exclusive, world-wide license to publish the Supplemental Material files. Please contact the corresponding author directly for reuse.
