# Supplementary material for: The metallophosphoesterase Rv0805 regulates carbon flux and cell envelope homeostasis during growth of mycobacteria in propionate
Source: J Bacteriol. 2026 Feb 27;208(3):e00571-25. doi: 10.1128/jb.00571-25 (PMC13001264; doi:10.1128/jb.00571-25)
Supplement: Supplemental figures — Figures S1 to S5. [file jb.00571-25-s0001.pdf]

Supplementary Figures

A

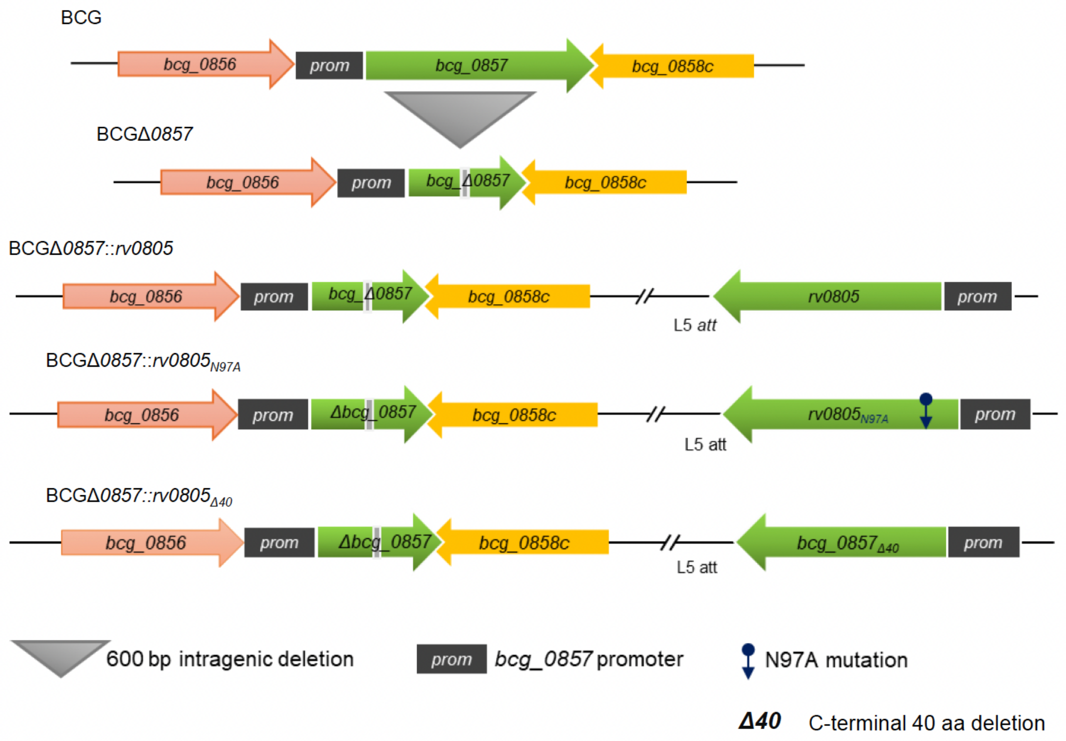

Figure S1. Schematic of strain construction.

Bcg_0857 was deleted from *M. bovis* BCG (BCG) to generate BCGΔ*0857* and complemented with either wild-type *rv0805*, $rv0805_{N97A}$, or $rv0805_{\Delta 40}$ under the native *rv0805* promoter at the L5 *att* site.

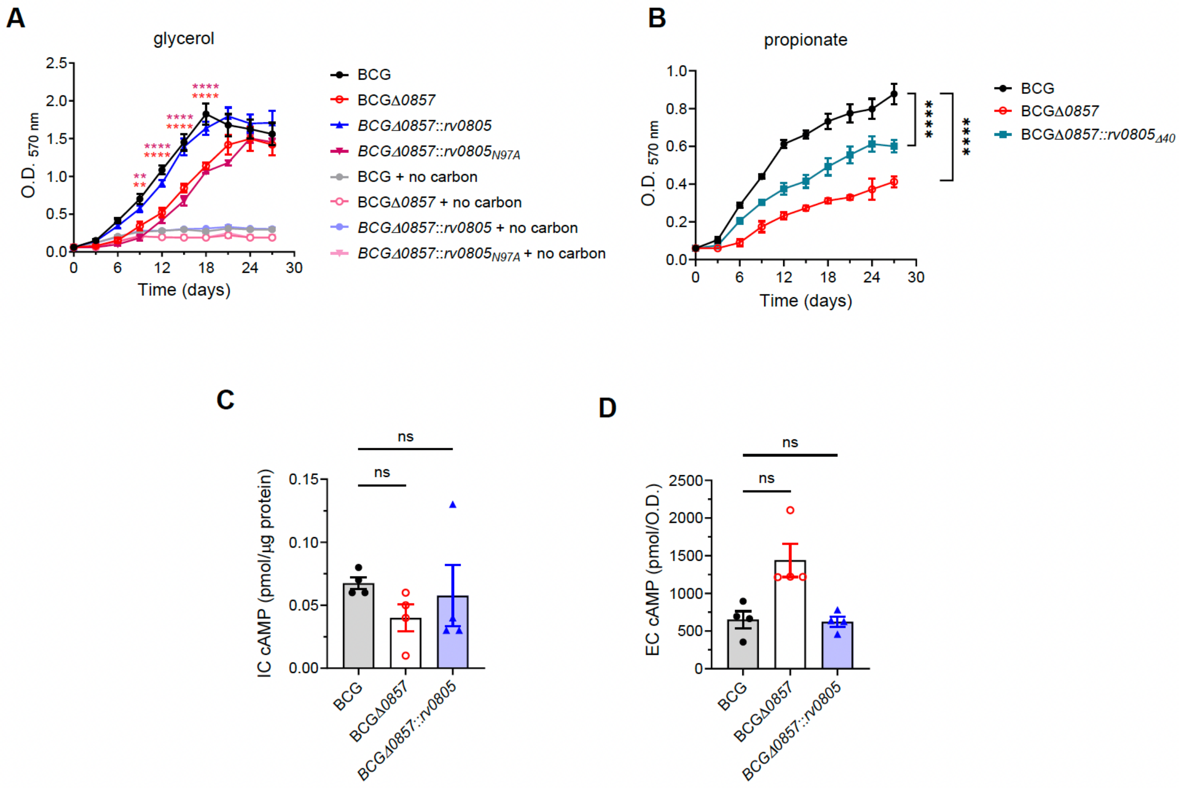

Figure S2. Growth analysis in glycerol.

Growth curves in minimal medium with (**A**) glycerol, or no carbon source ($n \geq 4$), and (**B**) propionate. (**C**) Intracellular (IC) and (**D**) extracellular (EC) cAMP levels in propionate-grown cultures (n=4). For **A-D**, results show mean ± SEM from biological replicates. Statistical significance was determined by two-way ANOVA with Dunnett's multiple comparison test for **A** and **B**; by one-way ANOVA with Tukey's multiple comparison test for **C** and **D**. **$p < 0.01$; ****$p < 0.0001$; ns, not significant.

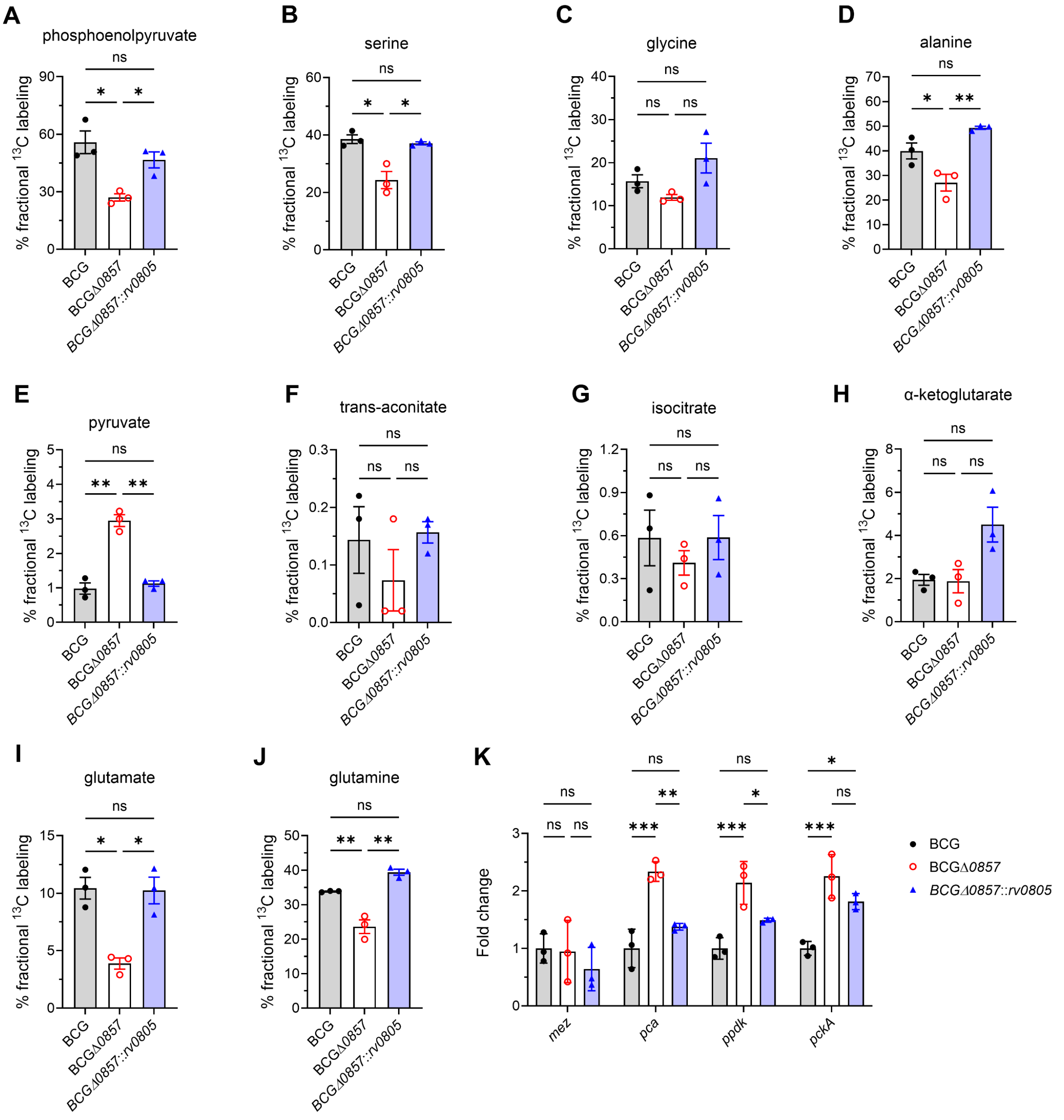

Figure S3. Fractional ^{13}C labeling of central carbon metabolites. ^{13}C-label incorporation from [U-^{13}C] propionate into CCM metabolites (**A**) phosphoenolpyruvate, (**B**) serine, (**C**) glycine, (**D**) alanine, (**E**) pyruvate, (**F**) trans-aconitate, (**G**) isocitrate, (**H**) α-ketoglutarate, (**I**) glutamate, and (**J**) glutamine reveals altered labeling patterns in BCGΔ*0857* (n=3). (**K**) QRT-PCR analysis of anaplerotic node genes *mez*, *pca*, *ppdk*, and *pckA* in propionate (n=3). For **A**-**K**, results show mean ± SEM from biological replicates. Statistical significance was determined by one-way ANOVA with Tukey's multiple comparison test for **A**-**J**, and by two-way ANOVA with Tukey's multiple comparison test for **K**. *p < 0.05; **p < 0.01; ***p < 0.001; ns, not significant.

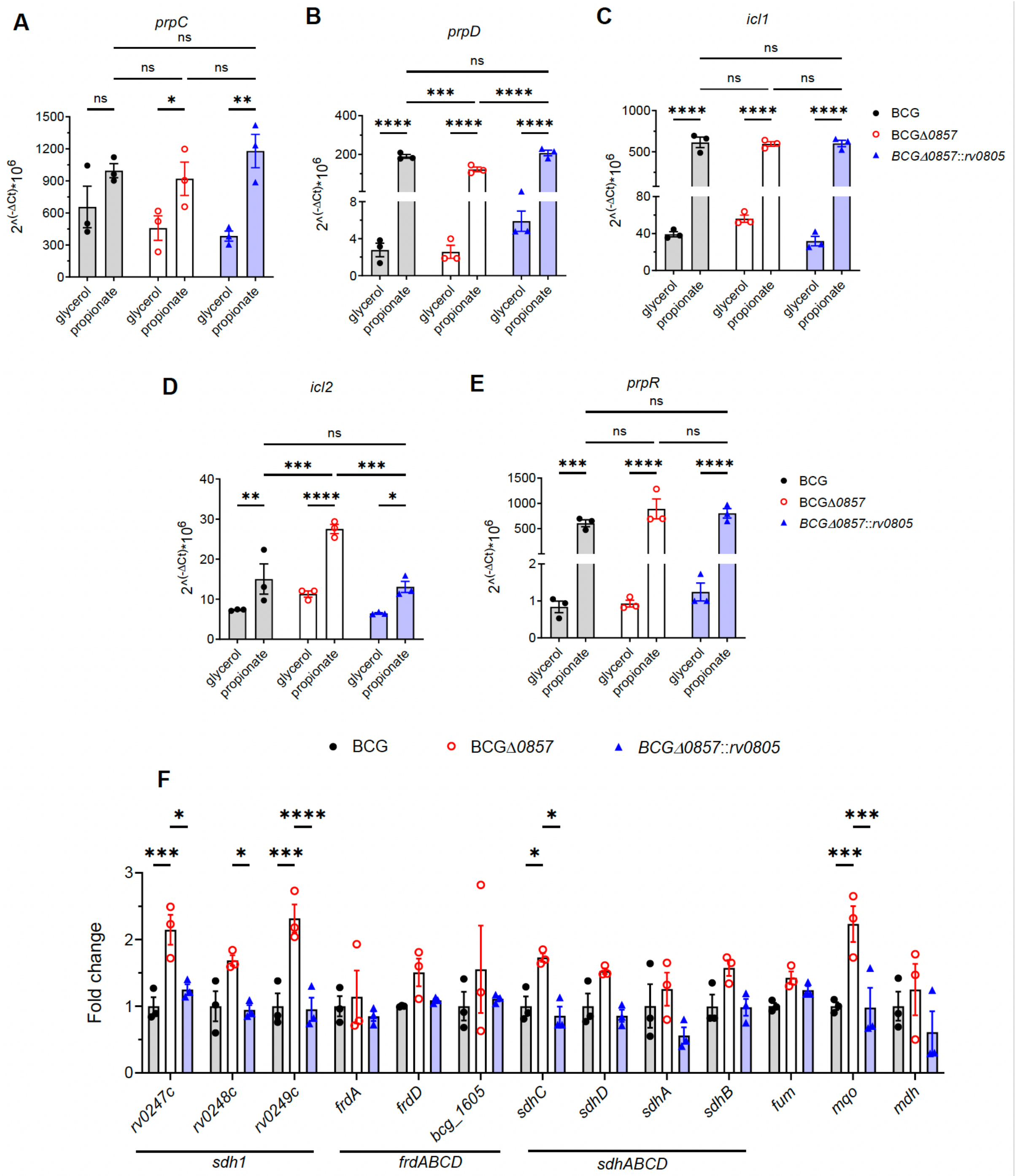

Figure S4. Analysis of the expression of MCC pathway genes.

QRT-PCR analysis of MCC pathway genes (**A**) *prpC*, (**B**) *prpD*, (**C**) *icl1*, and (**D**) *icl2* and their regulators (**E**) *prpR* in glycerol and propionate (n=3). (**F**) QRT-PCR of other MCC pathway genes (n=3). For A-E, results show mean ± SEM from biological replicates, and statistical significance was determined by two-way ANOVA with Tukey's multiple comparison test. In **F**, asterisks are used for only comparisons, where the p-value is less than 0.05. *p < 0.05; **p < 0.01; ***p < 0.001; ****p < 0.0001; ns, not significant. MCC: methylcitrate cycle.

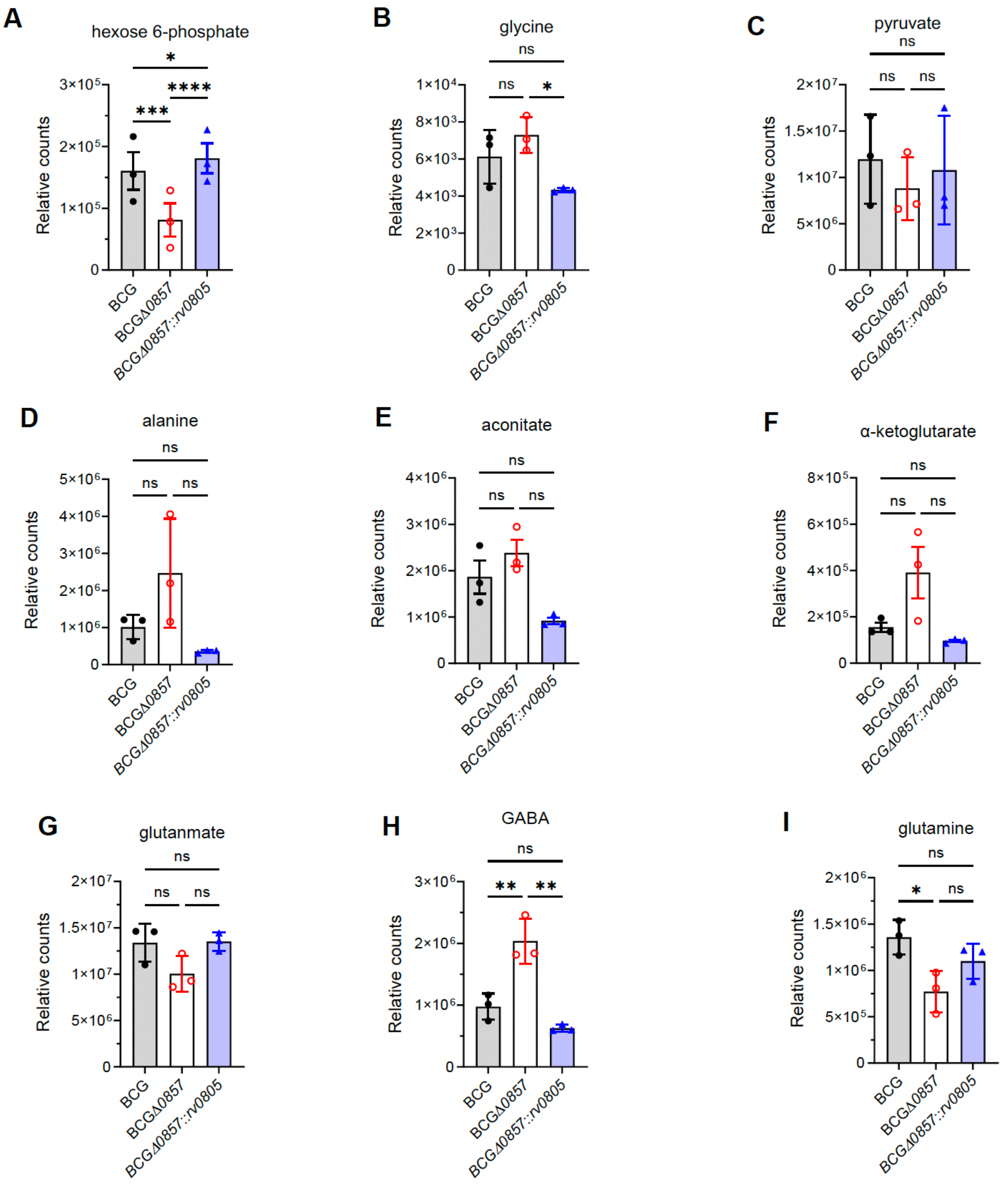

Figure S5. Broader metabolic consequences of *bcg_0857* deletion on central carbon metabolites.

Steady-state levels of central carbon metabolites (**A**) hexose 6-phosphate, (**B**) glycine, (**C**) pyruvate, (**D**) alanine, (**E**) aconitate, (**F**) α-ketoglutarate, (**G**) glutamate, (**H**) GABA, and (**I**) glutamine in propionate (n=3). For **A-I**, results show mean ± SEM from biological replicates, and statistical significance was determined by one-way ANOVA with Tukey's multiple comparison test. *$p < 0.05$; **$p < 0.01$; ****$p < 0.0001$; ****$p < 0.0001$; ns, not significant.
